# Baseline and follow-up change of cholesterol levels predict dementia risk and progression in older adults: a U-shaped relationship

**DOI:** 10.1186/s13195-025-01910-8

**Published:** 2025-11-26

**Authors:** Pai-Yi Chiu, Hsi-Hsien Chou, Chih-Li Lin, Hsin-Hua Li, Tzu-Yu Chen, Hong-Ming Chen, Hsin-Te Chang

**Affiliations:** 1 Department of Neurology, Show Chwan Memorial Hospital, Changhua City, Changhua, Taiwan; 2 Department of Smart Computing and Applied Mathematics, College of Science, Tunghai University, Taichung, Taiwan; 3 Department of Neurology, Chung Shan Medical University Hospital, College of Medicine, Chung Shan Medical University, Taichung, Taiwan; 4 Institute of Medicine, College of Medicine, Chung Shan Medical University, Taichung, Taiwan; 5 Department of Exercise Health Science, College of Sport Industry, National Taiwan University of Sport, Taichung, Taiwan; 6 General Education Center, National Taiwan University of Sport, Taichung, Taiwan; 7 Department of Psychology, College of Science, Chung Yuan Christian University, Taoyuan, Taiwan; 8 Research Assistive Center, Show Chwan Memorial Hospital, Changhua City, Changhua, Taiwan; 9 Department of Applied Psychology, College of Social Sciences, Hsuan Chuang University, Hsinchu City, Hsinchu, Taiwan

**Keywords:** Cholesterol, Dementia, Low-density lipoprotein cholesterol, High-density lipoprotein cholesterol, Triglyceride

## Abstract

**Introduction:**

Previous studies on serum lipid levels and cognitive outcomes have shown inconsistent results, partly due to differences in timing of lipid assessment, cognitive status, and lack of longitudinal data. This study aimed to examine both baseline and longitudinal changes in lipid profiles in relation to dementia onset and cognitive progression across different stages of cognitive impairment.

**Methods:**

A retrospective cohort study was conducted using data from the History-Based Artificial Intelligent Clinical Dementia Diagnostic System (HAICDDS), a multicenter memory clinic registry in Taiwan. Among 2,452 adults aged ≥ 60 years, lipid levels (total cholesterol, low-density lipoprotein cholesterol [LDL-c], high-density lipoprotein cholesterol [HDL-c], and triglycerides [TG]) were assessed at baseline and follow-up. Participants were stratified into subjective cognitive decline, mild cognitive impairment, or dementia. Cox proportional hazards models were used to evaluate associations with incident dementia or cognitive progression.

**Results:**

U-shaped associations were observed between lipid levels and cognitive outcomes. After adjusting for demographic and vascular risk factors, both low baseline values and extreme reductions—particularly in HDL-c and TG—were significantly associated with increased risk of dementia onset or progression.

**Conclusion:**

Lipid instability, especially in HDL-c and TG, may serve as a marker of cognitive vulnerability. These findings suggest that longitudinal changes in serum cholesterol should be carefully monitored in older adults at risk of cognitive decline.

**Supplementary Information:**

The online version contains supplementary material available at 10.1186/s13195-025-01910-8.

## Introduction

The global burden of dementia continues to rise, with over 55 million people currently affected and projections exceeding 150 million by 2050 [1]. While vascular risk factors such as hypertension and diabetes have been well-established contributors to cognitive decline [2, 3], the role of lipid metabolism—particularly serum cholesterol—remains controversial [4–7].

Clinical guidelines have promoted the aggressive lowering of serum cholesterol to reduce cardiovascular disease risk [8, 9]. However, translating this paradigm into cognitive health has proven complex [7]. Recent findings suggest that lower cholesterol levels, especially in older adults, may not necessarily equate to better cognitive outcomes [4, 10, 11]. Early epidemiological research demonstrated that elevated midlife total cholesterol (TC) increases the risk of Alzheimer’s disease (AD) in later life [12]. Subsequent studies differentiated lipid subtypes, showing that higher low-density lipoprotein cholesterol (LDL-c) and triglycerides (TG) are associated with greater risk of dementia [13–15], whereas higher high-density lipoprotein cholesterol (HDL-c) has been consistently linked to reduced risk and better cognitive outcomes [16–18]; however, this interpretation remains controversial given accumulating contradictory findings.

Over the past five years, numerous studies have examined the relationship between serum cholesterol and its components and dementia risk or cognitive decline, but the results have been inconsistent [4, 19–22]. For example, Li et al. [23] found that both low and high TC were associated with increased dementia risk in a Chinese cohort, whereas Liu et al. [24] reported no significant association between TC and dementia in similar population. Regarding specific lipid components, contrary to the traditional views of the negative effects of high LDL-c and TG on cognitive functions, Katsumata et al. [25] observed better memory performances among older Japanese adults with elevated LDL-c levels. Similarly, Likewise, Zhou et al. [20] found that higher TG levels were protective against incident dementia among older adults in the UK. Furthermore, Huang et al. [26] reported a U-shaped relationship between serum HDL-c and dementia risk among older adults in the US. Similar discrepancies have been noted in other recent studies [27–30].

These inconsistencies likely stem from several methodological differences: timing of lipid measurement (midlife vs. late life), variation in cognitive outcome definitions (incident dementia, cognitive decline, specific domains), and differential adjustment for confounders such as vascular disease, APOE genotype, or medication use (including statins) [31–34]. Recent reviews have emphasized the need for more nuanced research designs that consider longitudinal lipid changes over time, not just baseline levels.

Biologically, cholesterol plays essential roles in neuronal membrane integrity, synaptic plasticity, and myelin formation, and serves as a precursor for neurosteroids involved in cognition and mood regulation [35–39]. The brain contains approximately one-quarter of the body’s total cholesterol, and impaired cholesterol transport or metabolism has been linked to β-amyloid aggregation, tau hyperphosphorylation, and neuroinflammation—key mechanisms in Alzheimer’s disease pathology [40–43]. Thus, declining peripheral cholesterol may reflect or exacerbate altered brain cholesterol homeostasis. Additionally, late-life reductions in serum cholesterol may indicate catabolic or frailty-related metabolic states associated with neurodegeneration [44, 45]. Together, these findings provide a mechanistic rationale for why lower cholesterol levels, particularly in late life, could be associated with increased dementia risk.

Despite these insights, most available evidence remains limited by cross-sectional design, small sample sizes, or inadequate statistical adjustment. In Taiwan, data specific to Asian populations are relatively scarce, though some emerging cohorts have begun exploring this relationship [46]. Given differences in dietary patterns, body composition, and cardiovascular risk profiles between Asian and Western populations, population-specific analyses are warranted.

This study aims to address several of these gaps. Using a large, well-characterized cohort of older adults in Taiwan, we analyzed both baseline lipid levels and their longitudinal changes in relation to dementia incidence among individuals with subjective cognitive decline (SCD) and mild cognitive impairment (MCI) and cognitive progression among individuals with dementia. By stratifying participants according to baseline cognitive status and controlling for vascular comorbidities, medication use, and demographic factors, we hypothesized that both low baseline cholesterol levels and longitudinal reductions in lipid profiles are associated with increased risk of dementia onset and cognitive progression, independent of vascular comorbidities and frailty.

## Methods

### Study population

We conducted a retrospective cohort study using data from older adults aged 60 and above, selected from a dataset built for the “History-Based Artificial Intelligent Clinical Dementia Diagnostic System (HAICDDS) Project” [47]. HAICDDS aims to have participants longitudinally receive regular serum laboratory examinations and cognitive assessments at each time of evaluation at the Neurology outpatient clinics across Taiwan to facilitate early management of dementia. Individuals with no difficulties in understanding the instructions of cognitive assessment and blood sampling were included in the dataset. All participants and their informants were interviewed by neuropsychologists with well training and requested to complete a neuropsychological assessment and surveys of daily living. Cognitive status was assessed by the Clinical Dementia Rating. Cognitive functions were assessed by the Cognitive Abilities Screening Instrument (CASI). Daily living functions were evaluated with History-based Artificial Intelligence Activities of Daily Living (HAIADL). Neuropsychiatric symptoms were assessed using the Neuropsychiatric Inventory (NPI).

**Fig. 1:**
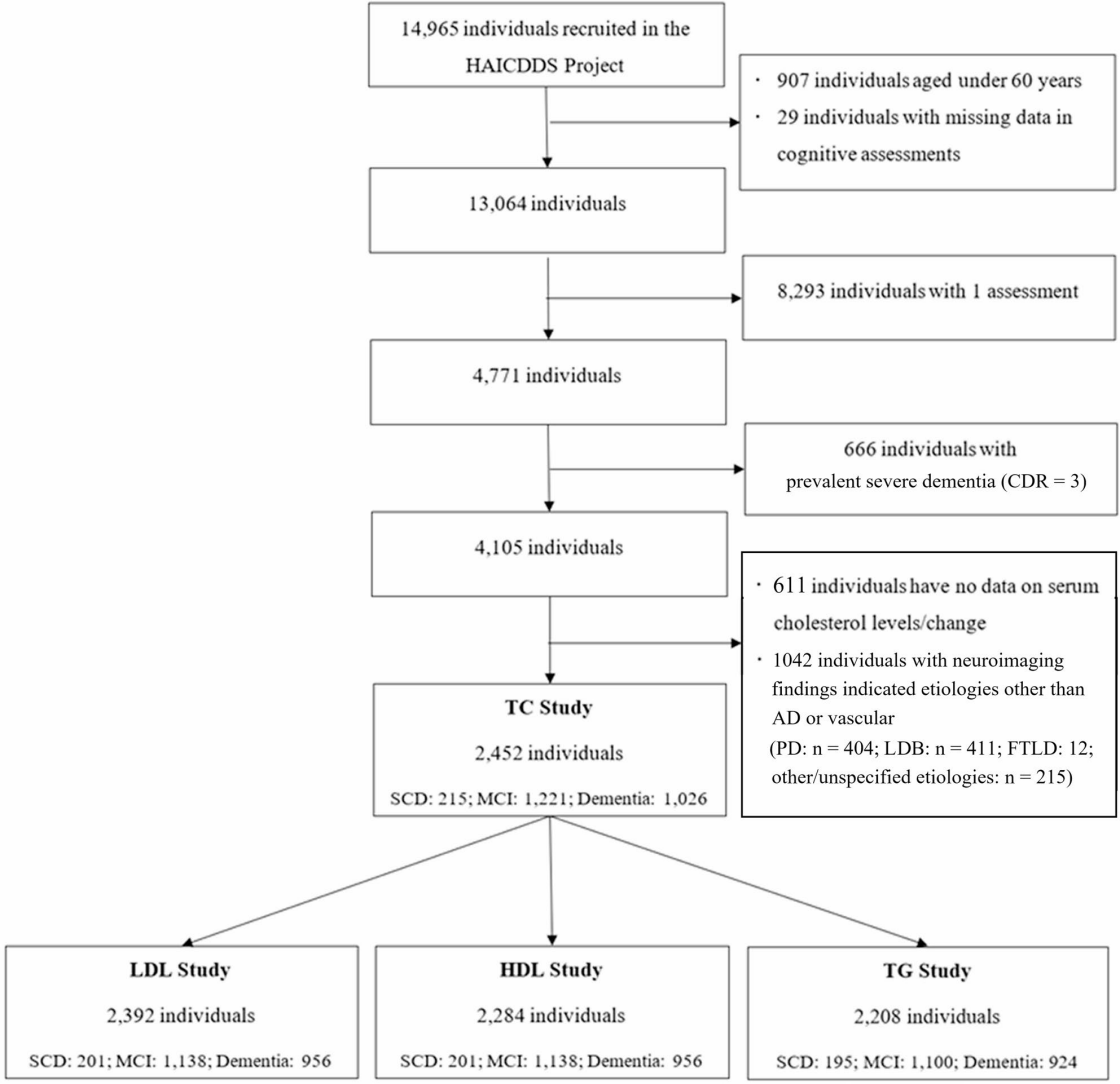
Selection process for study participants. Abbreviations. CDR: Clinical Dementia Rating. FTLD: Frontotemporal lobar degeneration. HAICDDS: History-Based Artificial Intelligent Clinical Dementia Diagnostic System. HDL: High-density lipoprotein cholesterol. LBD: Lewy body disease. LDL: Low-density lipoprotein cholesterol. MCI: Mild cognitive impairment. PD: Parkinson’s disease. SCD: Subjective cognitive decline. TC: Total cholesterol. TG: Triglyceride

As of December 25th, 2024, 14,965 participants have been included. Among them, 5,737 have been assessed at least twice. Data from 2,452 participants with at least two assessments in serum total cholesterol and cognitive function and aged over 60 years were extracted and analyzed in this study after excluding individuals aged under 60 years or with missing data in terms of demographics, serum total cholesterol levels, cerebrovascular diseases (CVD) and their risk factors, and drug treatment for the CVD (Fig. 1). The participants were enrolled in the project between April 2012 and June 2023. Each participant underwent baseline assessments of cognitive function, neuropsychiatric symptoms, and blood biomarkers at the time of enrollment. On average, participants completed 3.05 assessments (SD = 2.21, range = 2–7) over a mean follow-up period of 2.98 years (SD = 1.90, range = 0.5–5.17). The average interval between two examinations of cognitive functions and serum cholesterol was 0.88 years (SD = 1.12, range = 0.25–3.25 years).

Data concerning LDL-c, HDL-c, and TG were available among 2,392 (97.6%), 2,284 (93.1%), and 2,208 (90.0%) individuals, respectively, because of laboratory processing errors or incomplete records during data entry. Brain MRI, CT, or Tc-99 m-TRODAT SPECT was used to exclude marked etiologies other than AD or cerebrovascular (e.g., frontotemporal lobar degeneration, Parkinson’s disease, or Lewy body disease). Participants with incomplete cognitive evaluations were also excluded.

Baseline characteristics of excluded versus included participants are provided in Supplementary Table 1. In brief, excluded participants were younger, more educated, less frail, and with lower vascular burden, compared with the included sample.

All data were retrospectively and anonymously analyzed. The study was approved by the Committee for Medical Research Ethics of Show Chwan Memorial Hospital (SCMH_IRB No: IRB1081006), and the need for informed consent was waived.

### Serum cholesterol

Serum lipid levels including TC, LDL-c, HDL-c, and TG were measured at baseline and repeatedly during follow-up. TC and TG were directly measured using enzymatic methods. HDL-c was measured by the Roche HDL-c 3rd generation direct method. LDL-c was calculated by formula of Friedewald: LDL-c = TC – HDL-c – TG/5.0 (mg/dL). For individuals with TG ≥ 400 mg/dL, a revised Friedwald formula was used to better estimate LDL-c: LDL-c = TC - HDL-c - TG/8.0 (mg/dL). For each lipid parameter, we derived baseline values and calculated the mean change across visits, defined as the average of interval-to-interval differences in lipid levels (i.e., the mean of all pairwise visit-to-visit differences during follow-up). For each lipid parameter, both baseline levels and mean longitudinal changes were categorized into quartiles (Q1–Q4) to facilitate comparison across exposure ranges.

### Outcome variables

Cognitive status was classified into three categories: subjective cognitive decline (SCD), mild cognitive impairment (MCI), and dementia. SCD was defined as a global Clinical Dementia Rating (CDR) score of 0 or 0.5, a normal Cognitive Abilities Screening Instrument (CASI) score [48], and a normal History-Based Artificial Intelligence Activities of Daily Living score (HAI-ADL < 8.5) [49]. MCI was diagnosed according to established criteria [50] as impaired CASI performance adjusted for age and education, a normal HAI-ADL score, and a CDR of 0.5 with a sum of boxes < 4.5 [47]. Dementia was diagnosed according to the National Institute on Aging–Alzheimer’s Association criteria [51]. Cognitive progression was defined as an increase in CDR global score or functional decline on ADL at follow-up, as assessed by neuropsychologists [52]. Based on baseline and follow-up evaluations, participants were classified into four cognitive trajectory groups: (1) SMCI-stable (SMCI-S): participants with SCD or MCI at baseline who remained stable without conversion at follow-up; (2) SMCI with incident dementia (SMCI-D): participants with SCD or MCI at baseline who progressed to dementia, defined as an increase in CDR global score ≥ 1 or a functional decline on the HAI-ADL scale; (3) Dementia-stable (Dementia-S): participants with dementia at baseline who showed no further CDR or ADL deterioration during follow-up; (4) Dementia with progression (Dementia-D): participants with dementia at baseline who exhibited cognitive or functional worsening, defined as an increase in CDR global score or decline in ADL. In the main analyses, the SMCI-S group served as the reference for comparisons within the SMCI cohort SMCI-D, and Dementia-S group served as the reference for comparisons within the dementia cohort (i.e., versus Dementia-D).

### Covariates

Covariates were selected a priori using a causal diagram to control for baseline confounding while minimizing adjustment for potential mediators. Demographic covariates included age, sex, and years of education. Vascular comorbidities (hypertension, diabetes mellitus, coronary artery disease, cerebrovascular disease, and arrhythmia) were included as pre-existing conditions. Frailty was assessed using the Clinical Frailty Scale (CFS) by trained evaluators, and dichotomized as ≥ 5 vs. < 5 [53], and lipid-lowering medication use was abstracted from prescription records. Comorbidities were clinician-diagnosed from electronic medical records; frailty and functional measures were obtained using standardized assessments; and lipid levels were measured in certified clinical laboratories.

### Statistical analysis

Cox proportional hazards regression models were used to estimate hazard ratios (HRs) and 95% confidence intervals (CIs) for incident dementia and cognitive progression. All models were adjusted for the covariates listed above. The proportional hazards assumption was verified for all models (ps > 0.05). Sensitivity analyses were performed by excluding participants who initiated, discontinued, or substantially modified lipid-lowering therapy during follow-up. Mann–Whitney U tests were performed to compare HRs between Q1 and Q4 subgroups. We additionally tested an interaction term (cholesterol × frailty) to explore whether associations differed by frailty status. Inverse probability weighting was applied to account for potential bias introduced by data selection when predicting conversion to dementia or cognitive progression in dementia [43]. The weighting model incorporated baseline variables that differed between included and excluded participants (Supplementary Table 1). Comparisons of regression coefficients (*β*) between the original models and the models adjusted by inverse probability weighting were conducted using z-tests for equality of coefficients [43]. To further explore whether baseline lipid levels modified the associations between longitudinal cholesterol changes and dementia risk, we conducted stratified analyses within the SMCI cohort. Participants were divided into quartiles (Q1–Q4) according to their baseline serum cholesterol levels (TC: Q1: <146 mg/dL; Q2: 146–169 mg/dL; Q3: 169–195 mg/dL; Q4: >196 mg/dL; LDL-c: Q1: <79 mg/dL; Q2: 80–99 mg/dL; Q3: 100–123 mg/dL; Q4: >123 mg/dL; HDL-c: Q1: <40 mg/dL; Q2: 41–48 mg/dL; Q3: 49–61 mg/dL; Q4: >61 mg/dL; TG: Q1: <76 mg/dL; Q2: 76–104 mg/dL; Q3: 105–147 mg/dL; Q4: >147 mg/dL). Within each quartile, we modeled the hazard ratio of dementia incidence as a function of longitudinal cholesterol change using natural spline Cox models adjusted for covariates. This approach allowed visualization of potential non-linear relationships between TC change and dementia risk across different baseline cholesterol strata. HRs relative to optimal serum cholesterol levels were visualized using natural spline regression models implemented in R.

## Results

A total of 2,452 participants were included. Table 1 displays the demographic and clinical characteristics. Supplementary Tables 2 to 17 presents the comparisons of the demographic and clinical characteristics between individuals with low or high serum cholesterol at baseline and between individuals with large reduction or increase in serum cholesterol.

**Table 1:** Demographics and clinical characteristics of the participants

	SMCI-S	SMCI-D	Dementia-S	Dementia-D	Statistical comparisons
n	975	451	601	425	
MCI (%)	66.05 (644/975)	88.91 (401/451)	--	--	$\:{\chi\:}_{n=2452,\:df=1}^{2}$= 82.32, *p* < 0.001
Age (yr)	74.09 (7.81)^*abc*^	78.33 (7.46)^*ade*^	79.98 (7.68)^*bdf*^	81.18 (7.50)^*cef*^	*F*_*(3,2448)*_ = 119.77, *p* < 0.001
Follow-up duration (yr)	3.15 (1.98)^*abc*^	3.49 (2.01)^*ade*^	2.61 (1.84)^*bd*^	2.60 (1.81)^*ce*^	*F*_*(3,2448)*_ = 7813.44, *p* < 0.001
Educational level (yr)	6.18 (4.63)^*abc*^	4.85 (4.30)^*ae*^	4.45 (4.43)^*b*^	4.21 (4.34)^*ce*^	*F*_*(3,2448)*_ = 29.02, *p* < 0.001
Sex (% male)	45.54 (444/975)	45.45 (205/451)	39.10 (235/601)	45.88 (195/425)	$\:{\chi\:}_{n=2452,\:df=3}^{2}$= 7.79, *p* = 0.05
Hypertension (%)	73.33 (715/975)^*a*^	80.71 (364/451)^*ae*^	77.54 (466/601)	74.12 (315/425)^*e*^	$\:{\chi\:}_{n=2452,\:df=3}^{2}$= 10.82, *p* < 0.05
Diabetes mellitus (%)	41.13 (401/975)^*abc*^	54.32 (245/451)^*a*^	53.58 (322/601)^*b*^	52.94 (225/425)^*c*^	$\:{\chi\:}_{n=2452,\:df=3}^{2}$= 36.87, *p* < 0.001
Coronary artery disease (%)	12.31 (120/975)	14.86 (67/451)	10.32 (62/601)	12.71 (54/425)	$\:{\chi\:}_{n=2452,\:df=3}^{2}$= 4.96, *p* = 0.18
Cerebrovascular disease (%)	31.49 (307/975)	42.57 (192/451)^*d*^	50.58 (304/601)^*d*^	55.29 (235/425)	$\:{\chi\:}_{n=2452,\:df=3}^{2}$= 92.99, *p* < 0.001
Arrhythmia (%)	15.59 (152/975)	20.18 (91/451)	14.64 (88/601)	14.82 (63/425)	$\:{\chi\:}_{n=2452,\:df=3}^{2}$= 7.21, *p* = 0.07
Hypercholesterolemia (%)	59.59 (581/975)^*bc*^	54.10 (244/451)^*e*^	48.92 (294/601)^*b*^	47.06 (200/425)^*ce*^	$\:{\chi\:}_{n=2452,\:df=3}^{2}$= 26.70, *p* < 0.001
Anti-hypertensive (%)	55.59 (542/975)	56.76 (256/451)	51.75 (311/601)	54.35 (231/425)	$\:{\chi\:}_{n=2452,\:df=3}^{2}$= 3.22, *p* = 0.36
Anti-diabetic (%)	26.15 (255/975)^*abc*^	33.70 (152/451)^*a*^	31.78 (191/601)^*b*^	33.41 (142/425)^*c*^	$\:{\chi\:}_{n=2452,\:df=3}^{2}$= 12.99, *p* < 0.01
Anti-platelets (%)	46.67 (455/975)	50.11 (226/451)	44.59 (268/601)	50.12 (213/425)	$\:{\chi\:}_{n=2452,\:df=3}^{2}$= 4.70, *p* = 0.20
Anti-coagulants (%)	10.26 (100/975)	14.41 (65/451)	10.98 (66/601)	12.00 (51/425)	$\:{\chi\:}_{n=2452,\:df=3}^{2}$= 5.50, *p* = 0.14
Anti-lipid agents (%)	49.74 (485/975)^*bc*^	47.45 (214/451)^*de*^	34.28 (206/601)^*b*^	37.41 (159/425)^*cde*^	$\:{\chi\:}_{n=2452,\:df=3}^{2}$= 45.56, *p* < 0.001
CASI (maximum score = 100)	74.39 (16.03)^*abc*^	53.03 (19.85)^*ade*^	42.56 (21.06)^*bdf*^	35.32 (20.49)^*cef*^	*F*_*(3,2448)*_ = 581.99, *p* < 0.001
HAI-ADL (maximum score = 43)	4.24 (4.01)^*abc*^	11.96 (7.29)^*ade*^	17.51 (6.26)^*bdf*^	19.59 (6.56)^*cef*^	*F*_*(3,2448)*_ = 1013.00, *p* < 0.001
NPI-SB (maximum score = 144)	4.23 (6.43)^*abc*^	7.97 (10.22)^*ae*^	8.98 (11.37)^*bf*^	10.17 (10.42)^*cef*^	*F*_*(3,2448)*_ = 55.94, *p* < 0.001
CDR-SB (maximum score = 18)	1.72 (1,82)^*abc*^	5.43 (3.59)^*ade*^	8.76 (3.43)^*bdf*^	10.02 (3.43)^*cef*^	*F*_*(3,2448)*_ = 1122.32, *p* < 0.001
CFS (maximum = 7)	1.97 (1.13)^*abc*^	3.43 (1.73)^*ade*^	4.60 (1.60)^*bdf*^	5.08 (1.57)^*cef*^	*F*_*(3,2448)*_ = 639.82, *p* < 0.001
TC (mg/dL)	155.98 (31.63)^*abc*^	142.93 (29.13)^*ad*^	152.00 (33.60)^*bdf*^	143.00 (35.57)^*cf.*^	*F*_*(3,2448)*_ = 25.49, *p* < 0.001
TC change (mg/dL)	−2.93 (24.14)	−3.14 (22.26)	−4.11 (23.93)	−3.69 (22.70)	*F*_*(3,2448)*_ = 0.49, *p* = 0.69
LDL-c (mg/dL)	88.75 (29.97)^*ac*^	78.14 (24.84)^*ad*^	87.40 (28.72)^*bdf*^	78.99 (29.73)^*cf.*^	*F*_*(3,2388)*_ = 22.86, *p* < 0.001
LDL-c change (mg/dL)	−2.84 (24.49)	−2.15 (21.61)	−3.32 (23.04)	−2.45 (21.06)	*F*_*(3,2388)*_ = 0.29, *p* = 0.84
HDL-c (mg/dL)	53.20 (16.05)^*bc*^	51.48 (16.24)	50.24 (16.67)^*b*^	49.56 (15.84)^*c*^	*F*_*(3,2380)*_ = 6.38, *p* < 0.001
HDL-c change (mg/dL)	0.49 (8.46)^*ac*^	−0.43 (9.56)^*a*^	0.35 (7.95)^*f*^	−1.13 (8.87)^*cf.*^	*F*_*(3,2380)*_ = 5.38, *p* < 0.01
TG (mg/dL)	115.59 (75.31)^*ac*^	106.34 (63.71)^*ad*^	117.03 (67.98)^*df*^	105.13 (62.70)^*cf.*^	*F*_*(3,2204)*_ = 4.20, *p* < 0.01
TG change (mg/dL)	−4.92 (80.92)	−1.36 (34.92)	−1.74 (48.67)	−1.16 (37.64)	*F*_*(3,2204)*_ = 0.87, *p* = 0.46

*CASI* Cognitive Assessment Screening Instrument, *CDR-SB* Clinical Dementia Rating-Sum of boxes, *CFS* Clinical Frailty Scale, *Dementia-D* Individuals with progressive dementia, *Dementia-S* Individuals with dementia who were not progressive, *HAI-ADL* History-Based Artificial Intelligence-Activities of Daily Living, *HDL-c* High-density lipoprotein cholesterol, *LDL-c* Low-density lipoprotein cholesterol, *MCI* Mild cognitive impairment, *NPI-SB* Neuropsychiatric Inventory-Sum of boxes, *SMCI-D* Subjective or mild cognitive impairment individuals who converted to dementia, *SMCI-S* Stable subjective or mild cognitive impairment individuals, *TC* Total cholesterol, *TG* Triglyceride

Numbers are denoted as mean (SD) or proportion (number)

The superscripts indicate statistically significant differences (*p* < 0.05) in the variable between the subgroups

^ a^: SMCI-S SMCI-D

^b^: SMCI-S Dementia-S

^c^: SMCI-S Dementia-D

^d^: SMCI-D Dementia-S

^e^: SMCI-D Dementia-D

^f^: Dementia-S Dementia-D

**Fig. 2:**
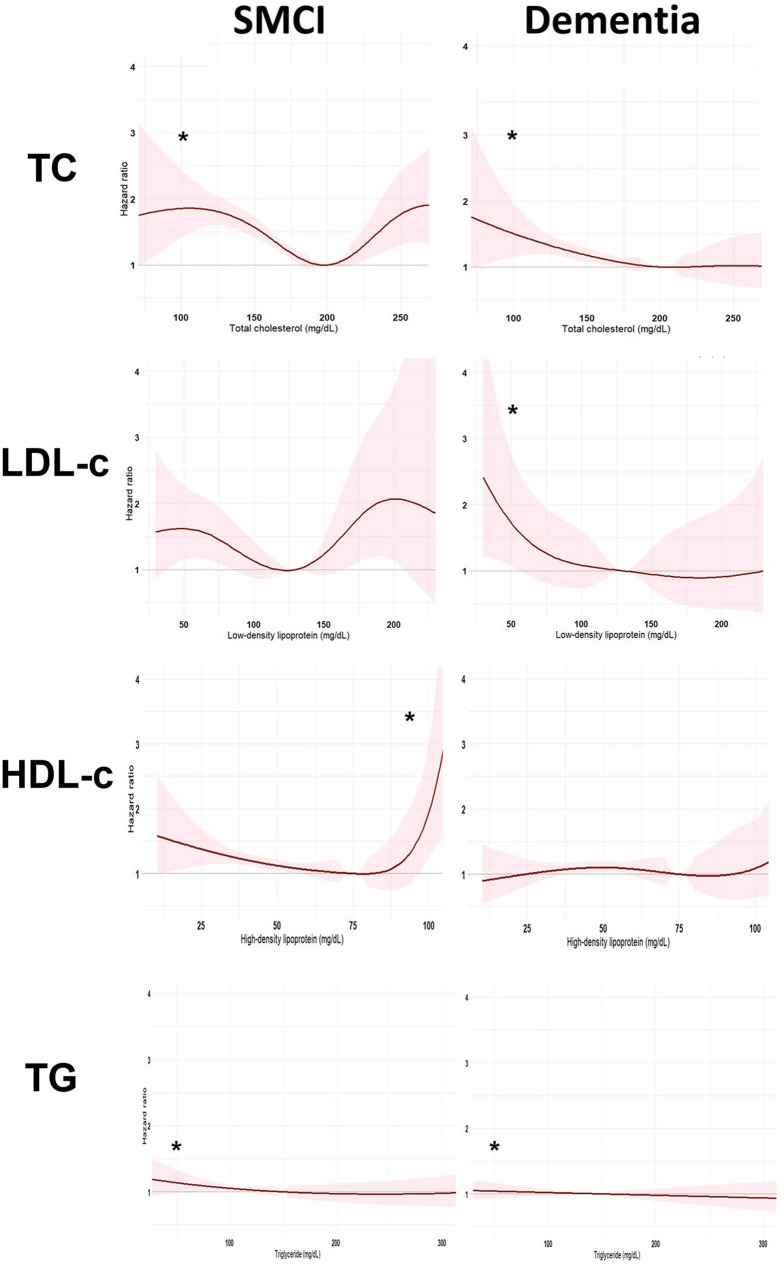
Risk of dementia onset and progression across levels of baseline cholesterol. *Note*. Asterisks indicate statistically significant differences in risk between individuals in the lowest (Q1) and highest (Q4) quartiles. The position of each asterisk reflects which group (Q1 or Q4) had the higher associated risk. Abbreviation. SMCI: participants with subjective or mild cognitive impairment at baseline; Dementia: participants with dementia at baseline

**Fig. 3:**
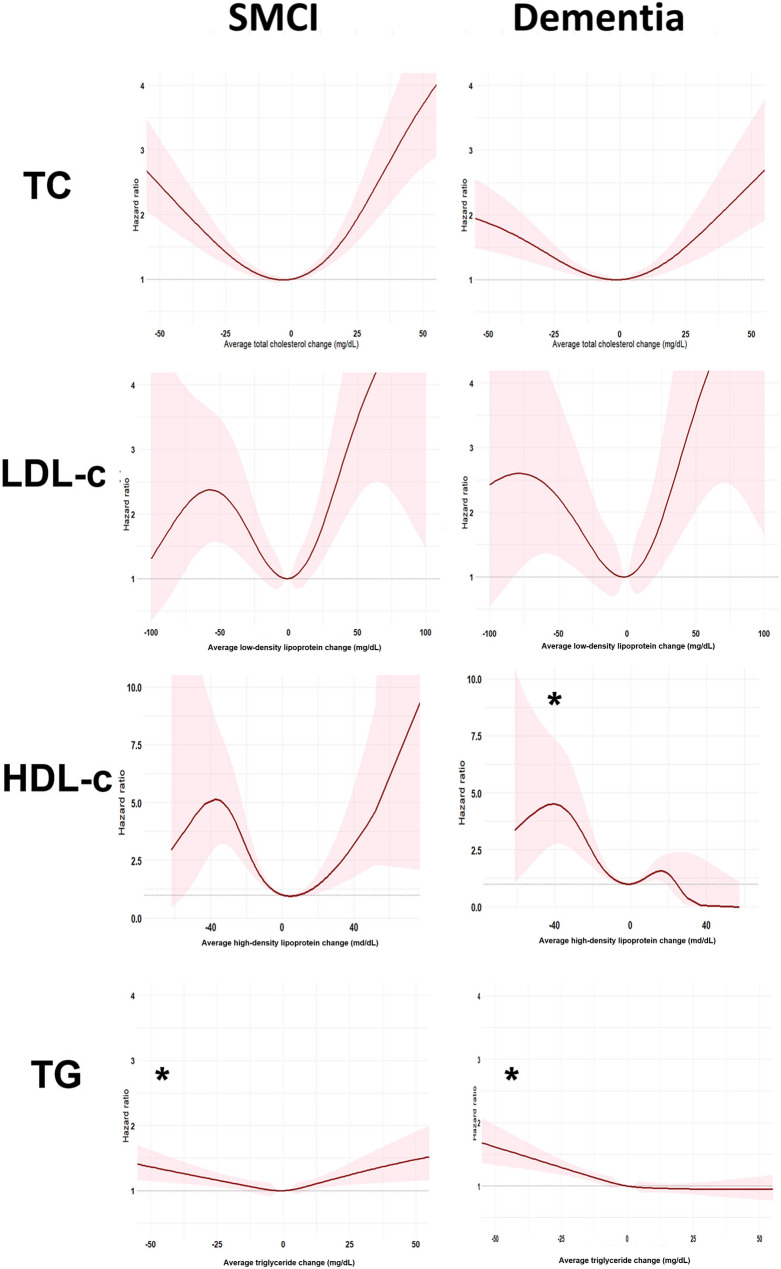
Risk of dementia onset and progression across levels of cholesterol change. Note and abbreviation are the same as those used in Fig. 2

Figures 2 and 3 display the association of serum cholesterol and hazard ratios of incident dementia or cognitive progression. Baseline low TC and TG levels were associated with higher risks for incident dementia and cognitive progression (SMCI: TC: median HR: 1.69 [95% CI: 1.40–2.51] vs. 1.34 [1.04–3.59], p < 0.001; TG: median HR: 1.16 [1.00–1.71] vs. 1.03 [0.99–2.12]; dementia: TC: median HR: 1.42 [1.26–1.78] vs. 1.27 [0.81–1.34], p < 0.001; TG: median HR: 1.28 [1.10–1.98] vs. 0.96 [0.95–1.79], p < 0.001). Baseline low LDL-c levels were associated with higher risks for cognitive progression among patients with prevalent dementia (median HR: 1.47 [1.20–2.56] vs. 0.91 [0.90–0.94], p < 0.001). Baseline high HDL-c was associated with higher risks of incident dementia among the individuals with SMCI (median HR: 1.35 [1.24–1.58] vs. 1.25 [0.99–5.64], p < 0.001). Relationships between longitudinal changes in cholesterol levels and risks of incident dementia and cognitive progression conformed to U-shaped like relationships. There were no differences in the risks of incident dementia and cognitive progression among the individuals with SMCI (p = 0.07–0.49) or dementia with larger changes in cholesterols, with the exceptions of higher risks of incident dementia among SMCI individuals with greater reduction in TG (median HR: 1.53 [0.10–2.50] vs. 1.43 [0.74–1.68], p < 0.001) and of cognitive progression among individuals with dementia with larger reduction in HDL-c (median HR: 1.64 [1.10–4.81] vs. 1.28 [0.01–1.98], p < 0.001) and TG (1.68 [0.59–2.50] vs. 0.93 [0.92–10.00]) (Supplementary Table 18).

A significant interaction was observed between TG levels and frailty status (baseline: *p* = 0.02; change in TG levels: *p* = 0.03). Specifically, participants with both lower levels of or greater reduction in TG and higher frailty (CFS ≥ 5) exhibited a markedly increased risk of incident dementia (baseline: HR = 2.08, *[*1.13–3.94*]*; change: HR = 3.24, *[*1.45–4.62*]*) and cognitive progression (baseline: HR = 1.99, *[*1.08–2.13*]*; HR = 4.14, *[*1.65–7.93*]*) compared with non-frail individuals (baseline: HR = 1.02, *[*0.89–2.01*]*; change: HR = 1.00, *[*0.88–1.12*]*) or individuals with higher TG levels (baseline: HR = 0.87, *[*0.60–1.13*]*; change: HR = 0.76, *[*0.53–1.01*]*). No significant interactions were found for TC, LDL-c, or HDL-c.

No statistically significant differences were observed in the associations between incident dementia or cognitive progression and the predictor variables when using the original weights versus inverse probability weighting (all *p*s > 0.05).

In stratified analyses by baseline cholesterol quartiles among the SMCI cohort, the association between longitudinal cholesterol change and dementia risk demonstrated a consistent U-shaped pattern across most quartiles (Supplementary Figs. 1–4), indicating that both large increases and decreases in choleseterol were associated with higher dementia risk regardless of baseline cholesterol levels.

## Discussion

Our findings demonstrate a robust U-shaped association between serum cholesterol and cognitive outcomes, consistent with a growing number of recent longitudinal studies [16, 20, 54]. These results challenge the assumption that lower cholesterol levels are universally beneficial and suggest that the relationship between cholesterol and cognition may vary across individuals and disease stages.

Notably, our study is among the few to concurrently assess both baseline lipid levels and their longitudinal changes. Most prior studies evaluated a single time point or average level of cholesterol across time, thereby failing to capture lipid trajectories. For instance, a US-based analysis found no significant baseline association but did not account for change over time [30]. Our dual approach suggests that lipid reductions, particularly in HDL-c and TG, may be associated with or indicative of cognitive deterioration. Importantly, our study is also among the few to encompass multiple stages of cognitive impairment, from subjective cognitive decline to mild cognitive impairment and dementia, allowing for a more nuanced understanding of lipid–cognition associations across the disease continuum.

Our subgroup analyses further clarified prior inconsistencies. In the SMCI cohort, we observed stronger associations of TC and HDL-c with cognitive outcomes, whereas in the dementia cohort, TG reductions were most strongly associated with worsening. This may reflect stage-dependent metabolic vulnerability, a nuance not explored in earlier studies.

Among participants, baseline TC levels in the lowest quartile (Q1) were associated with significantly higher risk of incident dementia or cognitive progression compared to the highest quartile (Q4). This finding indicates that extremely low TC levels were associated with higher dementia risk compared with elevated levels. Additionally, TC change analyses showed that both extreme increases and decreases in TC were associated with elevated risk (HRs > 2.0), with no evidence that a decline was safer than an increase. This pattern may reflect underlying metabolic or neurobiological processes, such as dysregulation of cholesterol homeostasis [55]. These findings highlight the importance of cholesterol homeostasis, and not just absolute levels, in maintaining cognitive health.

In the SMCI group, individuals with extremely high baseline HDL-c had significantly increased risk compared to those in the highest quartile. A recent study with large cohort also reported that high HDL-c might be detrimental for cognitive functions [54]. Experimental studies have suggested that extremely high HDL-c could be linked to increased oxidative stress and inflammation [56]. Interestingly, among individuals already diagnosed with dementia, a sharp decline in HDL-c was associated with significantly elevated risk of cognitive deterioration. While HDL-c is traditionally seen as “protective,” these results suggest that extreme elevations or rapid reductions—particularly in later stages of cognitive impairment—may be associated with loss of compensatory mechanisms or ongoing neurodegeneration.

Similar to the findings in a recent prospective study with a large sample [20], we found both SMCI and dementia groups exhibited higher risk associated with low baseline TG levels compared to high TG levels. TG measured in late life are more likely to be influenced by contemporaneous lifestyle behaviors, nutrition, and health conditions [20]. In contrast, studies reporting associations between serum TG and incident dementia commonly involve individuals at midlife and may reflect longer-term exposure to hypertriglycedemia.[57, 58]. More notably, extreme reductions in TG were linked to significantly greater hazard than extreme increases in both groups. These findings are consistent with recent evidence. Our stratified analyses further revealed that frailty modified the association between TG levels and dementia risk. Individuals with both low TG and higher frailty (CFS ≥ 5) had substantially greater risk of dementia and cognitive progression, suggesting that lipid-related vulnerability may be compounded by systemic frailty [20]. This finding highlights the importance of considering frailty status when interpreting late-life TG levels, and may partly explain inconsistencies in prior studies that did not adjust for frailty. However, biological mechanisms underpinning the potential relationship between low TG and high risk of dementia and/or cognitive decline remain unknown. A cross-sectional study reported that levels of 2 components consisting of long-chain polyunsaturated fatty acid-containing TG were significantly lower in patients with MCI or AD compared to individuals with normal cognitive functions [59]. Future studies are needed to further understand the mechanisms of neuroprotective effects of serum TG.

Our study benefits from a large, clinically heterogeneous sample, rigorous cognitive classification across the spectrum of impairment—including subjective cognitive decline, mild cognitive impairment, and dementia—and robust statistical control for key confounders, including vascular comorbidities and lipid-lowering medication use. Nonetheless, several limitations warrant consideration. First, the retrospective design limits causal inference, and the relatively short follow-up period may preclude the detection of long-term cognitive trajectories. The association between lipid reduction and dementia risk may partly reflect reverse causality, as early cognitive decline can result in dietary changes and weight loss that lower cholesterol levels [60]. In addition, most participants included in the registry had only baseline data without subsequent follow-up, which restricted our ability to evaluate longitudinal trajectories of serum cholesterol and cognitive progression in a larger and more heterogeneous population. Moreover, individuals with prevalent severe dementia at entry were excluded because their advanced cognitive and functional impairment made reliable neuropsychological testing and longitudinal comparison infeasible. This exclusion criterion, however, may introduce a degree of survival or selection bias, as participants with more advanced disease or frailty were less likely to be followed, potentially underestimating associations with rapid decline. Second, although mixed-effects modeling could in theory better capture individual lipid trajectories, the limited number of repeated measurements per participant (mean 3.05, median 2) and the focus on time-to-event outcomes favored the use of Cox regression for this study. This approach provides interpretable estimates of dementia risk associated with overall lipid change. Future study with longer follow-up duration should investigate the effects of mixed-effects modeling on capturing individual lipid trajectories and the effects on relationship between serum cholesterol and cognitive progression. Third, due to sample size constraints, we were unable to stratify analyses by clinically meaningful subgroups such as sex or detailed categories of lipid-lowering medication use, which may have introduced residual heterogeneity. Fourth, APOE genotype data were not available, precluding adjustment for this important genetic risk factor. In addition, routine and complete body mass index (BMI) data were not available for the entire cohort. To avoid substantial loss of sample size and selection bias, we retained BMI outside the primary adjustment set, and therefore the role of metabolic or nutritional factors as potential intermediates warrants further investigation. Fifth, unmeasured confounding from factors such as systemic inflammation may persist despite statistical adjustments. Sixth, as our sample was drawn from a clinical setting, findings may not be generalizable to community-based or cognitively normal populations. Seventh, we defined longitudinal changes as mean interval-to-interval differences, rather than time-standardized slopes; while this approach captures instability across visits, alternative definitions (e.g., mixed-effect slopes) may yield different interpretations. Eighth, lipid change was defined as the mean interval-to-interval difference, which reflects the overall direction and magnitude of longitudinal change rather than intra-individual variability. Future studies with more evenly spaced follow-up assessments could explore alternative definitions (e.g., intra-individual SD or coefficient of variation) to further validate these findings. Finally, although we adjusted for age, comorbidities, and frailty in Cox models, we acknowledge that residual baseline imbalances may remain. Future studies using randomized designs are warranted.

## Conclusions

These findings have important implications for geriatric lipid management. In contrast to the cardiovascular emphasis on aggressive lipid lowering, our findings underscore the need to further investigate how lipid levels relate to cognitive vulnerability in older adults. Longitudinal monitoring of lipid profiles, particularly HDL-c and TG, may offer prognostic utility and help tailor intervention strategies.

In older Taiwanese adults, both low and declining cholesterol—particularly HDL-c and TG—were associated with increased dementia risk and cognitive decline. These findings highlight the importance of further evaluating lipid-cognition relationships in geriatric populations.

## Supplementary Information

Supplementary Material 1

## Data Availability

Data available on request due to privacy/ethical restrictions. Requests to access these datasets should be directed to H‑T C, changht@cycu.edu.tw.
