# Supplementary material for: Baseline and follow-up change of cholesterol levels predict dementia risk and progression in older adults: a U-shaped relationship
Source: Alzheimers Res Ther. 2025 Nov 26;17:273. doi: 10.1186/s13195-025-01910-8 (PMC12751808; doi:10.1186/s13195-025-01910-8)
Supplement: Supplementary file 1 — Supplementary Material 1 [file 13195_2025_1910_MOESM1_ESM.docx]

**Supplementary information**

**Baseline and longitudinal cholesterol levels predict dementia risk and progression**

**in older adults: A U-shaped relationship**

**Supplementary Table 1.** Demographics and clinical characteristics between participants included and excluded in this study

|  | Included individuals | Excluded individuals | Statistical comparisons |
| --- | --- | --- | --- |
| n | 2452 | 1653 |  |
| MCI (%) | 42.46 (1041/2452) | 27.77 (459/1653) | $\chi_{n=4105, df=1}^{2}$ = 91.85, *p* < 0.001 |
| Age (yr) | 78.40 (7.66) | 75.76 (10.13) | *F_(1,4103)_* = 90.11, *p* < 0.001 |
| Follow-up duration (yr) | 2.96 (1.99) | 2.61 (1.47) | *F_(1,4103)_* = 37.38, *p* < 0.001 |
| Educational level (yr) | 4.92 (4.40) | 5.62 (4.85) | *F_(1,4103)_* = 23.00, *p* < 0.001 |
| Sex (% male) | 44.00 (1079/2452) | 42.11 (696/1653) | $\chi_{n=4105, df=1}^{2}$ = 1.45, *p* = 0.23 |
| Hypertension (%) | 75.85 (1860/2452) | 55.35 (915/1653) | $\chi_{n=4105, df=1}^{2}$ = 189.50, *p* < 0.001 |
| Diabetes mellitus (%) | 48.65 (1193/2452) | 36.90 (610/1653) | $\chi_{n=4105, df=1}^{2}$ = 55.36, *p* < 0.001 |
| Coronary artery disease (%) | 12.36 (303/2452) | 7.74 (128/1653) | $\chi_{n=4105, df=1}^{2}$ = 48.64, *p* < 0.001 |
| Cerebrovascular disease (%) | 42.33 (1038/2452) | 32.67 (540/1653) | $\chi_{n=4105, df=1}^{2}$ = 38.98, *p* < 0.001 |
| Arrhythmia (%) | 16.07 (394/2452) | 8.59 (142/1653) | $\chi_{n=4105, df=1}^{2}$ = 48.64, *p* < 0.001 |
| Hypercholesterolemia (%) | 53.79 (1319/2452) | 37.39 (618/1653) | $\chi_{n=4105, df=1}^{2}$ = 106.64, *p* < 0.001 |
| Anti-hypertensive (%) | 54.65 (1340/2452) | 38.66 (639/1653) | $\chi_{n=4105, df=1}^{2}$ = 101.14, *p* < 0.001 |
| Anti-diabetic (%) | 30.18 (740/2452) | 20.33 (336/1653) | $\chi_{n=4105, df=1}^{2}$ = 49.56, *p* < 0.001 |
| Anti-platelets (%) | 47.39 (1162/2452) | 23.59 (390/1653) | $\chi_{n=4105, df=1}^{2}$ = 237.79, *p* < 0.001 |
| Anti-coagulants (%) | 11.50 (282/2452) | 5.69 (94/1653) | $\chi_{n=4105, df=1}^{2}$ = 40.11, *p* < 0.001 |
| Anti-lipid agents (%) | 43.39 (1064/2452) | 24.26 (401/1653) | $\chi_{n=4105, df=1}^{2}$ = 157.50, *p* < 0.001 |
| CASI (maximum score = 100) | 52.00 (17.92) | 53.26 (28.82) | *F_(1,4102)_* = 2.98, *p* = 0.08 |
| HAI-ADL (maximum score = 43) | 13.33 (10.06) | 12.58 (9.96) | *F_(1,4103)_* = 5.58, *p* < 0.05 |
| NPI-SB (maximum score = 144) | 13.33 (9.99) | 6.80 (9.17) | *F_(1,4103)_* = 450.42, *p* < 0.001 |
| CDR-SB (maximum score = 18) | 7.84 (3.01) | 6.19 (5.47) | *F_(1,4103)_* = 153.96, *p* < 0.001 |
| CFS (maximum = 7) | 6.48 (3.00) | 3.65 (2.30) | *F_(1,4103)_* = 1053.49, *p* < 0.001 |
| TC (mg/dL) | 148.48 (30.02) | 167.37 (37.98) | *F_(1,3685)_* = 314.73, *p* < 0.001 |
| TC change (mg/dL) | -3.47 (23.65) | -2.54 (22.89) | *F_(1,3685)_* = 1.57, *p* = 0.21 |
| LDL-c (mg/dL) | 83.32 (30.26) | 99.81 (32.51) | *F_(1,3617)_* = 275.97, *p* < 0.001 |
| LDL-c change (mg/dL) | -2.69 (25.40) | -0.93 (20.68) | *F_(1,3617)_* = 5.48, *p* < 0.05 |
| HDL-c (mg/dL) | 51.12 (17.82) | 50.41 (16.03) | *F_(1,3589)_* = 1.70, *p* = 0.19 |
| HDL-c change (mg/dL) | -0.18 (10.02) | -0.03 (7.99) | *F_(1,3589)_* = 0.26, *p* = 0.61 |
| TG (mg/dL) | 111.02 (73.21) | 124.17 (80.45) | *F_(1,3212)_* = 23.39, *p* < 0.001 |
| TG change (mg/dL) | -2.30 (83.23) | -1.13 (90.62) | *F_(1,3212)_* = 0.18, *p* = 0.67 |
| Abbreviations are the same as those used in Table 1. | | | |

**Supplementary Table 2.** Demographics and clinical characteristics of the SMCI participants with low or high serum TC at baseline examination

|  | 1^st^ Quartile | 4^th^ Quartile | Statistical comparisons |  |
| --- | --- | --- | --- | --- |
| n | 337 | 383 |  |  |
| MCI (%) | 84.87 (286/337) | 82.24 (315/383) | $\chi_{n=720, df=1}^{2}$ = 0.89, *p* = 0.34 |  |
| Age (yr) | 75.51 (7.51) | 73.96 (7.62) | *t_(df = 718)_* = 2.74, *p* < 0.01 |  |
| Follow-up duration (yr) | 3.07 (1.94) | 3.29 (1.93) | *t_(df = 718)_* = 1.53, *p* = 0.13 |  |
| Educational level (yr) | 5.85 (4.74) | 5.57 (4.61) | *t_(df = 718)_* = 0.80, *p* = 0.42 |  |
| Sex (% male) | 45.70 (154/337) | 45.70 (175/383) | $\chi_{n=720, df=1}^{2}$ = 0, *p* = 0.99 |  |
| Hypertension (%) | 78.05 (263/337) | 67.89 (260/383) | $\chi_{n=720, df=1}^{2}$ = 9.30, *p* < 0.01 |  |
| Diabetes mellitus (%) | 43.92 (148/337) | 68.93 (264/383) | $\chi_{n=720, df=1}^{2}$ = 45.82, *p* < 0.001 |  |
| Coronary artery disease (%) | 15.44 /52/337) | 12.28 (47/383) | $\chi_{n=720, df=1}^{2}$ = 82.32, *p* < 0.001 |  |
| Cerebrovascular disease (%) | 48.67 (164//337) | 32.64 (125/383) | $\chi_{n=720, df=1}^{2}$ = 1.51, *p* = 0.22 |  |
| Arrhythmia (%) | 20.18 (68/337) | 12.02 (46/383) | $\chi_{n=720, df=1}^{2}$ = 8.97, *p* < 0.01 |  |
| Anti-hypertensive (%) | 77.16 (260/337) | 65.28 (250/383) | $\chi_{n=720, df=1}^{2}$ = 82.32, *p* < 0.001 |  |
| Anti-diabetic (%) | 43.33 (146/337) | 23.76 (91/383) | $\chi_{n=720, df=1}^{2}$ = 31.07, *p* < 0.001 |  |
| Anti-platelets (%) | 57.87 (195/337) | 50.40 (193/383) | $\chi_{n=720, df=1}^{2}$ = 4.03, *p* < 0.05 |  |
| Anti-coagulants (%) | 18.40 (62/337) | 10.19 (39/383) | $\chi_{n=720, df=1}^{2}$ = 10.03, *p* < 0.002 |  |
| Anti-lipid agents (%) | 54.31 (183/337) | 54.47 (209/383) | $\chi_{n=720, df=1}^{2}$ = 0.01, *p* = 0.99 |  |
| CASI (maximum score = 100) | 68.88 (15.99) | 69.57 (16.94) | *t_(df = 718)_* = 0.56, *p* = 0.58 |  |
| HAI-ADL (maximum score = 43) | 9.89 (7.25) | 7.71 (6.62) | *t_(df = 718)_* = 4.22, *p* < 0.001 |  |
| NPI-SB (maximum score = 144) | 6.88 (9.64) | 7.00 (10.55) | *t_(df = 718)_* = 3.65, *p* < 0.001 |  |
| CDR-SB (maximum score = 18) | 5.06 (3.96) | 4.20 (3.82) | *t_(df = 718)_* = 2.97, *p* < 0.01 |  |
| CFS (maximum = 7) | 3.21 (1.70) | 2.75 (1.69) | *t_(df = 718)_* = 3.62, *p* < 0.001 |  |
| TC change | 8.24 (20.03) | -16.13 (28.42) | *t_(df = 718)_* = 13.13, *p* < 0.001 |  |
| CASI: Cognitive Assessment Screening Instrument; CDR-SB: Clinical Dementia Rating-Sum of boxes; CFS: Clinical Frailty Scale; Dementia-D: Individuals with progressive dementia; Dementia-S: Individuals with dementia who were not progressive; HAI-ADL: History-Based Artificial Intelligence-Activities of Daily Living; HDL-c: High-density lipoprotein cholesterol; LDL-c: Low-density lipoprotein cholesterol; MCI: Mild cognitive impairment; NPI-SB: Neuropsychiatric Inventory-Sum of boxes; SMCI-D: Subjective or mild cognitive impairment individuals who converted to dementia; SMCI-S: Stable subjective or mild cognitive impairment individuals; TC: Total cholesterol; TG: Triglyceride.  Numbers are denoted as mean (SD) or proportion (number) | | | | |

**Supplementary Table 3.** Demographics and clinical characteristics of the SMCI participants with low or high serum LDL-c at baseline examination

|  | 1^st^ Quartile | 4^th^ Quartile | Statistical comparisons |  |
| --- | --- | --- | --- | --- |
| n | 292 | 325 |  |  |
| MCI (%) | 84.25 (246/292) | 90.15 (293/325) | $\chi_{n=617, df=1}^{2}$ = 4.86, *p* < 0.05 |  |
| Age (yr) | 75.99 (7.60) | 73.86 (7.43) | *t_(df = 615)_* = 3.52, *p* < 0.001 |  |
| Follow-up duration (yr) | 2.99 (1.49) | 2.94 (1.44) | *t_(df = 615)_* = 0.42, *p* = 0.67 |  |
| Educational level (yr) | 5.47 (4.69) | 5.69 (4.55) | *t_(df = 615)_* = 0.59, *p* = 0.55 |  |
| Sex (% male) | 46.24 (135/292) | 39.08 (127/325) | $\chi_{n=617, df=1}^{2}$ = 3.22, *p* = 0.07 |  |
| Hypertension (%) | 78.09 (228/292) | 68.00 (221/325) | $\chi_{n=617, df=1}^{2}$ = 7.34, *p* < 0.01 |  |
| Diabetes mellitus (%) | 56.17 (164/292) | 34.16 (111/325) | $\chi_{n=617, df=1}^{2}$ = 29.28, *p* < 0.001 |  |
| Coronary artery disease (%) | 17.13 (50/292) | 14.16 (46/325) | $\chi_{n=617, df=1}^{2}$ = 1.03, *p* = 0.31 |  |
| Cerebrovascular disease (%) | 34.25 (100/292) | 26.16 (85/325) | $\chi_{n=617, df=1}^{2}$ = 4.80, *p* < 0.05 |  |
| Arrhythmia (%) | 19.18 (56/292) | 13.24 (43/325) | $\chi_{n=617, df=1}^{2}$ = 4.04, *p* < 0.05 |  |
| Anti-hypertensive (%) | 47.27 (138/292) | 27.08 (88/325) | $\chi_{n=617, df=1}^{2}$ = 26.99, *p* < 0.001 |  |
| Anti-diabetic (%) | 61.31 (179/292) | 54.16 (176/325) | $\chi_{n=617, df=1}^{2}$ = 3.22, *p* = 0.08 |  |
| Anti-platelets (%) | 61.31 (179/292) | 54.16 (176/325) | $\chi_{n=617, df=1}^{2}$ = 3.22, *p* = 0.08 |  |
| Anti-coagulants (%) | 19.18 (56/292) | 10.16 (33/325) | $\chi_{n=617, df=1}^{2}$ = 10.15, *p* < 0.01 |  |
| Anti-lipid agents (%) | 38.02 (111/292) | 42.16 (137/325) | $\chi_{n=617, df=1}^{2}$ = 1.10, *p* = 0.29 |  |
| CASI (maximum score = 100) | 68.71 (15.53) | 70.21 (17.12) | *t_(df = 615)_* = 1.14, *p* = 0.26 |  |
| HAI-ADL (maximum score = 43) | 4.64 (3.64) | 3.83 (2.97) | *t_(df = 615)_* = 3.05, *p* < 0.01 |  |
| NPI-SB (maximum score = 144) | 4.28 (6.55) | 4.50 (5.99) | *t_(df = 615)_* = 0.44, *p* = 0.66 |  |
| CDR-SB (maximum score = 18) | 1.95 (1,64) | 1.65 (1.26) | *t_(df = 615)_* = 2.63, *p* < 0.01 |  |
| CFS (maximum = 7) | 2.08 (1.13) | 1.77 (0.95) | *t_(df = 615)_* = 8.41, *p* < 0.001 |  |
| LDL-c change | 11.82 (20.10) | -18.04 (26.77) | *t_(df = 615)_* = 15.53, *p* < 0.001 |  |
| CASI: Cognitive Assessment Screening Instrument; CDR-SB: Clinical Dementia Rating-Sum of boxes; CFS: Clinical Frailty Scale; Dementia-D: Individuals with progressive dementia; Dementia-S: Individuals with dementia who were not progressive; HAI-ADL: History-Based Artificial Intelligence-Activities of Daily Living; HDL-c: High-density lipoprotein cholesterol; LDL-c: Low-density lipoprotein cholesterol; MCI: Mild cognitive impairment; NPI-SB: Neuropsychiatric Inventory-Sum of boxes; SMCI-D: Subjective or mild cognitive impairment individuals who converted to dementia; SMCI-S: Stable subjective or mild cognitive impairment individuals; TC: Total cholesterol; TG: Triglyceride.  Numbers are denoted as mean (SD) or proportion (number) | | | | |

**Supplementary Table 4.** Demographics and clinical characteristics of the SMCI participants with low or high serum HDL-c at baseline examination

|  | 1^st^ Quartile | 4^th^ Quartile | Statistical comparisons |  |
| --- | --- | --- | --- | --- |
| n | 308 | 351 |  |  |
| MCI (%) | 90.26 (278/292) | 85.19 (299/325) | $\chi_{n=637, df=1}^{2}$ = 2.61, *p* = 0.11 |  |
| Age (yr) | 74.55 (7.57) | 74.89 (7.52) | *t_(df = 657)_* = 0.58, *p* = 0.56 |  |
| Follow-up duration (yr) | 3.01 (1.48) | 3.26 (1.52) | *t_(df = 657)_* = 2.14, *p* < 0.05 |  |
| Educational level (yr) | 5.94 (4.52) | 5.25 (4.42) | *t_(df = 657)_* = 1.98, *p* < 0.05 |  |
| Sex (% male) | 60.07 (185/308) | 28.21 (99/351) | $\chi_{n=659, df=1}^{2}$ = 67.90, *p* < 0.001 |  |
| Hypertension (%) | 78.25 (241/308) | 71.23 (250/351) | $\chi_{n=659, df=1}^{2}$ = 4.26, *p* < 0.05 |  |
| Diabetes mellitus (%) | 49.03 (151/308) | 33.05 (116/351) | $\chi_{n=659, df=1}^{2}$ = 17.38, *p* < 0.001 |  |
| Coronary artery disease (%) | 15.26 (47/308) | 11.12 (39/351) | $\chi_{n=659, df=1}^{2}$ = 2.49, *p* = 0.11 |  |
| Cerebrovascular disease (%) | 34.10 (105/308) | 24.22 (85/351) | $\chi_{n=659, df=1}^{2}$ = 7.80, *p* < 0.01 |  |
| Arrhythmia (%) | 15.26 (47/308) | 14.25 (50/351) | $\chi_{n=659, df=1}^{2}$ = 0.13, *p* = 0.71 |  |
| Anti-hypertensive (%) | 76.30 (235/308) | 70.09 (246/351) | $\chi_{n=659, df=1}^{2}$ = 3.21, *p* = 0.07 |  |
| Anti-diabetic (%) | 41.24 (127/308) | 25.08 (88/351) | $\chi_{n=659, df=1}^{2}$ = 19.50, *p* < 0.001 |  |
| Anti-platelets (%) | 61.04 (188/308) | 56.13 (197/351) | $\chi_{n=659, df=1}^{2}$ = 1.63, *p* = 0.20 |  |
| Anti-coagulants (%) | 13.32 (41/308) | 14.25 (50/351) | $\chi_{n=659, df=1}^{2}$ = 0.12, *p* = 0.73 |  |
| Anti-lipid agents (%) | 28.25 (87/308) | 37.04 (130/351) | $\chi_{n=659, df=1}^{2}$ = 5.74, *p* < 0.05 |  |
| CASI (maximum score = 100) | 66.12 (13.46) | 67.26 (16.42) | *t_(df = 657)_* = 3.52, *p* < 0.001 |  |
| HAI-ADL (maximum score = 43) | 4.39 (3.39) | 3.90 (3.03) | *t_(df = 657)_* = 3.52, *p* < 0.001 |  |
| NPI-SB (maximum score = 144) | 4.35 (6.10) | 4.21 (6.01) | *t_(df = 657)_* = 3.52, *p* < 0.001 |  |
| CDR-SB (maximum score = 18) | 1.85 (1.47) | 1.66 (1.41) | *t_(df = 657)_* = 3.52, *p* < 0.001 |  |
| CFS (maximum = 7) | 2.06 (1.18) | 1.85 (1.02) | *t_(df = 657)_* = 3.52, *p* < 0.001 |  |
| HDL-c change | 3.96 (9.41) | -3.78 (9.97) | *t_(df = 657)_* = 3.52, *p* < 0.001 |  |
| CASI: Cognitive Assessment Screening Instrument; CDR-SB: Clinical Dementia Rating-Sum of boxes; CFS: Clinical Frailty Scale; Dementia-D: Individuals with progressive dementia; Dementia-S: Individuals with dementia who were not progressive; HAI-ADL: History-Based Artificial Intelligence-Activities of Daily Living; HDL-c: High-density lipoprotein cholesterol; LDL-c: Low-density lipoprotein cholesterol; MCI: Mild cognitive impairment; NPI-SB: Neuropsychiatric Inventory-Sum of boxes; SMCI-D: Subjective or mild cognitive impairment individuals who converted to dementia; SMCI-S: Stable subjective or mild cognitive impairment individuals; TC: Total cholesterol; TG: Triglyceride.  Numbers are denoted as mean (SD) or proportion (number) | | | | |

**Supplementary Table 5.** Demographics and clinical characteristics of the SMCI participants with low or high serum TG at baseline examination

|  | 1^st^ Quartile | 4^th^ Quartile | Statistical comparisons |  |
| --- | --- | --- | --- | --- |
| n | 338 | 325 |  |  |
| MCI (%) | | 78.99 (267/338) | 90.15 (293/325) | $\chi_{n=663, df=1}^{2}$ =15.73, *p* < 0.001 |
| MCI (%) | 65.90 (203/338) | 70.15 (228/325) | $\chi_{n=663, df=1}^{2}$ = 0.07, *p* = 0.80 |  |
| Age (yr) | 74.88 (7.51) | 74.13 (7.54) | *t_(df = 661)_* = 1.28, *p* = 0.20 |  |
| Follow-up duration (yr) | 2.94 (1.50) | 2.98 (1.56) | *t_(df = 661)_* = 0.34, *p* = 0.74 |  |
| Educational level (yr) | 5.66 (4.32) | 5.56 (4.36) | *t_(df = 661)_* = 0.30, *p* = 0.77 |  |
| Sex (% male) | 51.19 (173/338) | 39.08 (127/325) | $\chi_{n=663, df=1}^{2}$ = 9.80, *p* < 0.01 |  |
| Hypertension (%) | 68.05 (230/338) | 82.16 (267/325) | $\chi_{n=663, df=1}^{2}$ = 17.57, *p* < 0.001 |  |
| Diabetes mellitus (%) | 35.21 (119/338) | 52.00 (169/325) | $\chi_{n=663, df=1}^{2}$ = 19.02, *p* < 0.001 |  |
| Coronary artery disease (%) | 12.14 (41/338) | 16.00 (52/325) | $\chi_{n=663, df=1}^{2}$ = 2.06, *p* = 0.15 |  |
| Cerebrovascular disease (%) | 29.29 (99/338) | 30.16 (98/325) | $\chi_{n=663, df=1}^{2}$ = 0.06, *p* = 0.81 |  |
| Arrhythmia (%) | 16.28 (55/338) | 15.08 (49/325) | $\chi_{n=663, df=1}^{2}$ = 0.18, *p* = 0.67 |  |
| Anti-hypertensive (%) | 68.05 (230/338) | 81.24 (264/325) | $\chi_{n=663, df=1}^{2}$ = 15.16, *p* < 0.001 |  |
| Anti-diabetic (%) | 27.22 (92/338) | 43.08 (140/325) | $\chi_{n=663, df=1}^{2}$ = 18.32, *p* < 0.001 |  |
| Anti-platelets (%) | 53.26 (180/338) | 59.08 (192/325) | $\chi_{n=663, df=1}^{2}$ = 2.28, *p* = 0.13 |  |
| Anti-coagulants (%) | 15.09 (51/338) | 13.34 (43/325) | $\chi_{n=663, df=1}^{2}$ = 0.47, *p* = 0.49 |  |
| Anti-lipid agents (%) | 28.11 (95/338) | 37.24 (121/325) | $\chi_{n=663, df=1}^{2}$ = 6.28, *p* < 0.05 |  |
| CASI (maximum score = 100) | 68.51 (14.25) | 66.13 (13.77) | *t_(df = 661)_* = 3.52, *p* < 0.001 |  |
| HAI-ADL (maximum score = 43) | 3.53 (2.54) | 3.92 (3.00) | *t_(df = 661)_* = 3.52, *p* < 0.001 |  |
| NPI-SB (maximum score = 144) | 4.90 (6.91) | 4.63 (5.98) | *t_(df = 661)_* = 3.52, *p* < 0.001 |  |
| CDR-SB (maximum score = 18) | 1.85 (1.47) | 1.66 (1.41) | *t_(df = 661)_* = 3.52, *p* < 0.001 |  |
| CFS (maximum = 7) | 1.77 (1.00) | 2.13 (1.25) | *t_(df = 661)_* = 3.52, *p* < 0.001 |  |
| TG change | 12.86 (24.14) | -42.95 (106.46) | *t_(df = 661)_* = 3.52, *p* < 0.001 |  |
| CASI: Cognitive Assessment Screening Instrument; CDR-SB: Clinical Dementia Rating-Sum of boxes; CFS: Clinical Frailty Scale; Dementia-D: Individuals with progressive dementia; Dementia-S: Individuals with dementia who were not progressive; HAI-ADL: History-Based Artificial Intelligence-Activities of Daily Living; HDL-c: High-density lipoprotein cholesterol; LDL-c: Low-density lipoprotein cholesterol; MCI: Mild cognitive impairment; NPI-SB: Neuropsychiatric Inventory-Sum of boxes; SMCI-D: Subjective or mild cognitive impairment individuals who converted to dementia; SMCI-S: Stable subjective or mild cognitive impairment individuals; TC: Total cholesterol; TG: Triglyceride.  Numbers are denoted as mean (SD) or proportion (number) | | | | |

**Supplementary Table 6.** Demographics and clinical characteristics of the participants with dementia and low or high serum TC at baseline examination

|  | 1^st^ Quartile | 4^th^ Quartile | Statistical comparisons |  |
| --- | --- | --- | --- | --- |
| n | 292 | 226 |  |  |
| Age (yr) | 80.13 (7.63) | 78.98 (7.44) | *t_(df = 376)_* = 1.72, *p* = 0.09 |  |
| Follow-up duration (yr) | 2.55 (1.73) | 2.60 (1.75) | *t_(df = 376)_* = 0.33, *p* = 0.75 |  |
| Educational level (yr) | 4.45 (4.33) | 4.12 (4.25) | *t_(df = 376)_* = 0.87, *p* = 0.39 |  |
| Sex (% male) | 48.29 (141/292) | 28.32 (64/226) | $\chi_{n=378, df=1}^{2}$ = 82.32, *p* < 0.001 |  |
| Hypertension (%) | 73.29 (214/292) | 74.34 (168/226) | $\chi_{n=378, df=1}^{2}$ = 82.32, *p* < 0.001 |  |
| Diabetes mellitus (%) | 57.20 (167/292) | 38.06 (86/226) | $\chi_{n=378, df=1}^{2}$ = 82.32, *p* < 0.001 |  |
| Coronary artery disease (%) | 12.33 (36/292) | 8.41 (19/226) | $\chi_{n=378, df=1}^{2}$ = 82.32, *p* < 0.001 |  |
| Cerebrovascular disease (%) | 54.11 (158/292) | 41.16 (93/226) | $\chi_{n=378, df=1}^{2}$ = 82.32, *p* < 0.001 |  |
| Arrhythmia (%) | 17.13 (50/292) | 11.07 (25/226) | $\chi_{n=378, df=1}^{2}$ = 82.32, *p* < 0.001 |  |
| Anti-hypertensive (%) | 70.21 (205/292) | 72.13 (163/226) | $\chi_{n=378, df=1}^{2}$ = 82.32, *p* < 0.001 |  |
| Anti-diabetic (%) | 44.18 (129/292) | 30.09 (68/226) | $\chi_{n=378, df=1}^{2}$ = 82.32, *p* < 0.001 |  |
| Anti-platelets (%) | 57.20 (167/292) | 51.33 (116/226) | $\chi_{n=378, df=1}^{2}$ = 82.32, *p* < 0.001 |  |
| Anti-coagulants (%) | 16.10 (47/292) | 9.30 (21/226) | $\chi_{n=378, df=1}^{2}$ = 82.32, *p* < 0.001 |  |
| Anti-lipid agents (%) | 47.27 (138/292) | 49.12 (111/226) | $\chi_{n=378, df=1}^{2}$ = 82.32, *p* < 0.001 |  |
| CASI (maximum score = 100) | 42.16 (19.41) | 42.70 (19.31) | *t_(df = 376)_* = 0.32, *p* = 0.75 |  |
| HAI-ADL (maximum score = 43) | 16.46 (5.68) | 15.63 (5.96) | *t_(df = 376)_* = 1.62, *p* = 0.11 |  |
| NPI-SB (maximum score = 144) | 10.30 (12.25) | 11.07 (12.90) | *t_(df = 376)_* = 0.69, *p* = 0.49 |  |
| CDR-SB (maximum score = 18) | 8.22 (3.04) | 8.29 (3.05) | *t_(df = 376)_* = 0.26, *p* = 0.80 |  |
| CFS (maximum = 7) | 4.44 (1.40) | 4.26 (1.44) | *t_(df = 376)_* = 1.43, *p* = 0.15 |  |
| TC change | 7.15 (20.88) | -19.04 (28.84) | *t_(df = 376)_* = 11.98, *p* < 0.001 |  |
| CASI: Cognitive Assessment Screening Instrument; CDR-SB: Clinical Dementia Rating-Sum of boxes; CFS: Clinical Frailty Scale; Dementia-D: Individuals with progressive dementia; Dementia-S: Individuals with dementia who were not progressive; HAI-ADL: History-Based Artificial Intelligence-Activities of Daily Living; HDL-c: High-density lipoprotein cholesterol; LDL-c: Low-density lipoprotein cholesterol; MCI: Mild cognitive impairment; NPI-SB: Neuropsychiatric Inventory-Sum of boxes; SMCI-D: Subjective or mild cognitive impairment individuals who converted to dementia; SMCI-S: Stable subjective or mild cognitive impairment individuals; TC: Total cholesterol; TG: Triglyceride.  Numbers are denoted as mean (SD) or proportion (number) | | | | |

**Supplementary Table 7.** Demographics and clinical characteristics of the participants with dementia and low or high serum LDL-c at baseline examination

|  | 1^st^ Quartile | 4^th^ Quartile | Statistical comparisons |  |
| --- | --- | --- | --- | --- |
| n | 209 | 169 |  |  |
| Age (yr) | 79.19 (7.20) | 78.71 (6.95) | *t_(df = 376)_* = 0.66, *p* = 0.51 |  |
| Follow-up duration (yr) | 2.69 (1.42) | 2.85 (1.48) | *t_(df = 376)_* = 1.07, *p* = 0.29 |  |
| Educational level (yr) | 4.51 (4.45) | 4.18 (4.39) | *t_(df = 376)_* = 0.72, *p* = 0.47 |  |
| Sex (% male) | 43.07 (90/209) | 30.18 (51/169) | $\chi_{n=378, df=1}^{2}$ = 6.63, *p* < 0.05 |  |
| Hypertension (%) | 78.47(164/209) | 78.11 (132/169) | $\chi_{n=378, df=1}^{2}$ = 0.01, *p* = 0.93 |  |
| Diabetes mellitus (%) | 60.29 (126/209) | 42.02 (71/169) | $\chi_{n=378, df=1}^{2}$ = 12.51, *p* < 0.001 |  |
| Coronary artery disease (%) | 54.07 (113/209) | 46.16 (78/169) | $\chi_{n=378, df=1}^{2}$ = 2.34, *p* = 0.13 |  |
| Cerebrovascular disease (%) | 13.40 (28/209) | 9.47 (16/169) | $\chi_{n=378, df=1}^{2}$ = 1.40, *p* = 0.24 |  |
| Arrhythmia (%) | 17.23 (36/209) | 13.01 (22/169) | $\chi_{n=378, df=1}^{2}$ = 1.27, *p* = 0.26 |  |
| Anti-hypertensive (%) | 47.37 (99/209) | 34.32 (58/169) | $\chi_{n=378, df=1}^{2}$ = 6.55, *p* < 0.05 |  |
| Anti-diabetic (%) | 67.47 (141/209) | 58.58 (99/169) | $\chi_{n=378, df=1}^{2}$ = 3.18, *p* = 0.07 |  |
| Anti-platelets (%) | 67.47 (141/209) | 58.58 (99/169) | $\chi_{n=378, df=1}^{2}$ = 3.18, *p* = 0.07 |  |
| Anti-coagulants (%) | 17.23 (36/209) | 12.43 (21/169) | $\chi_{n=378, df=1}^{2}$ = 1.68, *p* = 0.19 |  |
| Anti-lipid agents (%) | 39.24 (82/209) | 32.55 (55/169) | $\chi_{n=378, df=1}^{2}$ = 1.81, *p* = 0.18 |  |
| CASI (maximum score = 100) | 43.59 (19.52) | 45.15 (18.52) | *t_(df = 376)_* = 0.79, *p* = 0.43 |  |
| HAI-ADL (maximum score = 43) | 15.74 (5.53) | 15.29 (5.85) | *t_(df = 376)_* = 0.76, *p* = 0.44 |  |
| NPI-SB (maximum score = 144) | 9.45 (10.84) | 10.81 (11.50) | *t_(df = 376)_* = 1.18, *p* = 0.24 |  |
| CDR-SB (maximum score = 18) | 7.78 (3.02) | 7.90 (2.95) | *t_(df = 376)_* = 0.39, *p* = 0.70 |  |
| CFS (maximum = 7) | 4.20 (1.35) | 4.19 (1.46) | *t_(df = 376)_* = 0.07, *p* = 0.95 |  |
| LDL-c change | 7.71 (20.59) | -19.09 (27.22) | *t_(df = 376)_* = 10.89, *p* < 0.001 |  |
| CASI: Cognitive Assessment Screening Instrument; CDR-SB: Clinical Dementia Rating-Sum of boxes; CFS: Clinical Frailty Scale; Dementia-D: Individuals with progressive dementia; Dementia-S: Individuals with dementia who were not progressive; HAI-ADL: History-Based Artificial Intelligence-Activities of Daily Living; HDL-c: High-density lipoprotein cholesterol; LDL-c: Low-density lipoprotein cholesterol; MCI: Mild cognitive impairment; NPI-SB: Neuropsychiatric Inventory-Sum of boxes; SMCI-D: Subjective or mild cognitive impairment individuals who converted to dementia; SMCI-S: Stable subjective or mild cognitive impairment individuals; TC: Total cholesterol; TG: Triglyceride.  Numbers are denoted as mean (SD) or proportion (number) | | | | |

**Supplementary Table 8.** Demographics and clinical characteristics of the participants with dementia and low or high serum HDL-c at baseline examination

|  | 1^st^ Quartile | 4^th^ Quartile | Statistical comparisons |  |
| --- | --- | --- | --- | --- |
| n | 258 | 206 |  |  |
| Age (yr) | 79.26 (7.24) | 79.61 (7.04) | *t_(df = 376)_* = 0.52, *p* = 0.60 |  |
| Follow-up duration (yr) | 2.78 (1.49) | 2.69 (1.50) | *t_(df = 462)_* = 0.64, *p* = 0.52 |  |
| Educational level (yr) | 4.90 (4.34) | 4.28 (4.36) | *t_(df = 462)_* = 1.53, *p* = 0.13 |  |
| Sex (% male) | 55.04 (142/258) | 28.16 (58/206) | $\chi_{n=464, df=1}^{2}$ = 33.76, *p* < 0.001 |  |
| Hypertension (%) | 74.04 (191/258) | 70.39 (145/206) | $\chi_{n=464, df=1}^{2}$ = 96.95, *p* < 0.001 |  |
| Diabetes mellitus (%) | 57.37 (148/258) | 35.44 (73/206) | $\chi_{n=464, df=1}^{2}$ = 22.08, *p* < 0.001 |  |
| Coronary artery disease (%) | 14.35 (37/258) | 8.26 (17/206) | $\chi_{n=464, df=1}^{2}$ = 4.13, *p* < 0.05 |  |
| Cerebrovascular disease (%) | 46.13 (119/258) | 37.38 (77/206) | $\chi_{n=464, df=1}^{2}$ = 3.59, *p* = 0.06 |  |
| Arrhythmia (%) | 12.02 (31/258) | 13.11 (27/206) | $\chi_{n=464, df=1}^{2}$ = 0.12, *p* = 0.72 |  |
| Anti-hypertensive (%) | 74.04 (191/258) | 69.42 (143/206) | $\chi_{n=464, df=1}^{2}$ = 1.21, *p* = 0.27 |  |
| Anti-diabetic (%) | 45.35 (117/258) | 26.22 (54/206) | $\chi_{n=464, df=1}^{2}$ = 1.49, *p* = 0.22 |  |
| Anti-platelets (%) | 59.31 (153/258) | 53.40 (110/206) | $\chi_{n=464, df=1}^{2}$ = 1.63, *p* = 0.20 |  |
| Anti-coagulants (%) | 13.18 (34/258) | 11.17 (23/206) | $\chi_{n=464, df=1}^{2}$ = 0.43, *p* = 0.51 |  |
| Anti-lipid agents (%) | 25.20 (65/258) | 25.25 (52/206) | $\chi_{n=464, df=1}^{2}$ = 0.00, *p* = 0.99 |  |
| CASI (maximum score = 100) | 44.21 (18.62) | 46.44 (19.90) | *t_(df = 462)_* = 1.24, *p* = 0.21 |  |
| HAI-ADL (maximum score = 43) | 13.82 (7.19) | 12.24 (6.90) | *t_(df = 462)_* = 2.39, *p* < 0.05 |  |
| NPI-SB (maximum score = 144) | 9.13 (10.14) | 8.35 (11.46) | *t_(df = 462)_* = 0.78, *p* = 0.44 |  |
| CDR-SB (maximum score = 18) | 7.78 (3.02) | 7.90 (2.95) | *t_(df = 462)_* = 0.43, *p* = 0.67 |  |
| CFS (maximum = 7) | 3.95 (1.65) | 3.48 (1.58) | *t_(df = 462)_* = 3.11, *p* < 0.001 |  |
| HDL-c change | 4.04 (8.22) | -4.54 (11.69) | *t_(df = 462)_* = 9.27, *p* < 0.001 |  |
| CASI: Cognitive Assessment Screening Instrument; CDR-SB: Clinical Dementia Rating-Sum of boxes; CFS: Clinical Frailty Scale; Dementia-D: Individuals with progressive dementia; Dementia-S: Individuals with dementia who were not progressive; HAI-ADL: History-Based Artificial Intelligence-Activities of Daily Living; HDL-c: High-density lipoprotein cholesterol; LDL-c: Low-density lipoprotein cholesterol; MCI: Mild cognitive impairment; NPI-SB: Neuropsychiatric Inventory-Sum of boxes; SMCI-D: Subjective or mild cognitive impairment individuals who converted to dementia; SMCI-S: Stable subjective or mild cognitive impairment individuals; TC: Total cholesterol; TG: Triglyceride.  Numbers are denoted as mean (SD) or proportion (number) | | | | |

**Supplementary Table 9.** Demographics and clinical characteristics of the participants with dementia and low or high serum TG at baseline examination

|  | 1^st^ Quartile | 4^th^ Quartile | Statistical comparisons |  |
| --- | --- | --- | --- | --- |
| n | 257 | 237 |  |  |
| Age (yr) | 79.82 (7.36) | 78.69 (7.34) | *t_(df = 376)_* = 1.71, *p* = 0.09 |  |
| Follow-up duration (yr) | 2.66 (1.46) | 2.76 (1.46) | *t_(df = 492)_* = 0.76, *p* = 0.45 |  |
| Educational level (yr) | 4.59 (4.58) | 4.36 (4.50) | *t_(df = 492)_* = 0.56, *p* = 0.57 |  |
| Sex (% male) | 47.09 (121/257) | 34.18 (81/237) | $\chi_{n=494, df=1}^{2}$ = 82.32, *p* < 0.001 |  |
| Hypertension (%) | 46.31 (119/257) | 78.06 (185/237) | $\chi_{n=494, df=1}^{2}$ = 82.32, *p* < 0.001 |  |
| Diabetes mellitus (%) | 36.19 (93/257) | 63.30 (150/237) | $\chi_{n=494, df=1}^{2}$ = 82.32, *p* < 0.001 |  |
| Coronary artery disease (%) | 10.12 (26/257) | 9.29 (22/237) | $\chi_{n=494, df=1}^{2}$ = 82.32, *p* < 0.001 |  |
| Cerebrovascular disease (%) | 43.20 (111/257) | 49.37 (117/237) | $\chi_{n=494, df=1}^{2}$ = 82.32, *p* < 0.001 |  |
| Arrhythmia (%) | 16.35 (42/257) | 12.24 (29/237) | $\chi_{n=494, df=1}^{2}$ = 82.32, *p* < 0.001 |  |
| Anti-hypertensive (%) | 68.10 (175/257) | 75.11 (178/237) | $\chi_{n=494, df=1}^{2}$ = 82.32, *p* < 0.001 |  |
| Anti-diabetic (%) | 27.24 (70/257) | 50.22 (119/237) | $\chi_{n=494, df=1}^{2}$ = 82.32, *p* < 0.001 |  |
| Anti-platelets (%) | 59.15 (152/257) | 55.28 (131/237) | $\chi_{n=494, df=1}^{2}$ = 82.32, *p* < 0.001 |  |
| Anti-coagulants (%) | 15.18 (39/257) | 12.24 (29/237) | $\chi_{n=494, df=1}^{2}$ = 82.32, *p* < 0.001 |  |
| Anti-lipid agents (%) | 21.02 (54/257) | 29.12 (69/237) | $\chi_{n=494, df=1}^{2}$ = 82.32, *p* < 0.001 |  |
| CASI (maximum score = 100) | 48.68 (17.21) | 46.93 (18.67) | *t_(df = 492)_* = 1.08, *p* = 0.28 |  |
| HAI-ADL (maximum score = 43) | 15.77 (6.37) | 16.19 (6.88) | *t_(df = 492)_* = 0.71, *p* = 0.48 |  |
| NPI-SB (maximum score = 144) | 11.95 (12.71) | 10.48 (9.89) | *t_(df = 492)_* = 1.43, *p* = 0.16 |  |
| CDR-SB (maximum score = 18) | 1.60 (1.20) | 1.72 (1.34) | *t_(df = 492)_* = 1.05, *p* = 0.29 |  |
| CFS (maximum = 7) | 3.68 (1.45) | 3.42 (1.69) | *t_(df = 492)_* = 1.84, *p* = 0.07 |  |
| TG change | 17.29 (32.33) | -29.72 (92.86) | *t_(df = 492)_* = 7.63, *p* < 0.001 |  |
| CASI: Cognitive Assessment Screening Instrument; CDR-SB: Clinical Dementia Rating-Sum of boxes; CFS: Clinical Frailty Scale; Dementia-D: Individuals with progressive dementia; Dementia-S: Individuals with dementia who were not progressive; HAI-ADL: History-Based Artificial Intelligence-Activities of Daily Living; HDL-c: High-density lipoprotein cholesterol; LDL-c: Low-density lipoprotein cholesterol; MCI: Mild cognitive impairment; NPI-SB: Neuropsychiatric Inventory-Sum of boxes; SMCI-D: Subjective or mild cognitive impairment individuals who converted to dementia; SMCI-S: Stable subjective or mild cognitive impairment individuals; TC: Total cholesterol; TG: Triglyceride.  Numbers are denoted as mean (SD) or proportion (number) | | | | |

**Supplementary Table 10.** Demographics and clinical characteristics of the SMCI participants with larger change in serum TC

|  | 1^st^ Quartile | 4^th^ Quartile | Statistical comparisons |  |
| --- | --- | --- | --- | --- |
| n | 359 | 383 |  |  |
| MCI (%) | 87.20 (295/359) | 90.03 (341/383) | $\chi_{n=742, df=1}^{2}$ = 7.12, *p* < 0.01 |  |
| Age (yr) | 75.51 (7.61) | 73.96 (7.62) | *t_(df = 749)_* = 2.77, *p* < 0.01 |  |
| Follow-up duration (yr) | 3.07 (1.94) | 3.29 (1.97) | *t_(df = 749)_* = 1.25, *p* = 0.21 |  |
| Educational level (yr) | 5.85 (4.74) | 5.57 (4.61) | *t_(df = 749)_* = 3.52, *p* < 0.001 |  |
| Sex (% male) | 56.27 (202/359) | 31.08 (119/383) | $\chi_{n=742, df=1}^{2}$ = 47.93, *p* < 0.001 |  |
| Hypertension (%) | 78.28 (281/359) | 68.15 (261/383) | $\chi_{n=742, df=1}^{2}$ = 9.14, *p* < 0.001 |  |
| Diabetes mellitus (%) | 56.27 (202/359) | 31.08 (119/383) | $\chi_{n=742, df=1}^{2}$ = 47.93, *p* < 0.001 |  |
| Coronary artery disease (%) | 15.05 (54/359) | 12.02 (46/383) | $\chi_{n=742, df=1}^{2}$ = 1.46, *p* = 0.23 |  |
| Cerebrovascular disease (%) | 43.18 (155/359) | 42.04 (161/383) | $\chi_{n=742, df=1}^{2}$ = 0.10, *p* = 0.75 |  |
| Arrhythmia (%) | 20.06 (72/359) | 12.02 (46/383) | $\chi_{n=742, df=1}^{2}$ = 8.97, *p* < 0.01 |  |
| Anti-hypertensive (%) | 77.16 (277/359) | 65.02 (249/383) | $\chi_{n=742, df=1}^{2}$ = 13.25, *p* < 0.001 |  |
| Anti-diabetic (%) | 44.02 (158/359) | 24.03 (92/383) | $\chi_{n=742, df=1}^{2}$ = 33.15, *p* < 0.001 |  |
| Anti-platelets (%) | 49.03 (176/359) | 50.14 (192/383) | $\chi_{n=742, df=1}^{2}$ = 13.25, *p* < 0.001 |  |
| Anti-coagulants (%) | 18.11 (65/359) | 10.19 (39/383) | $\chi_{n=742, df=1}^{2}$ = 9.65, *p* < 0.01 |  |
| Anti-lipid agents (%) | 50.14 (180/359) | 50.14 (192/383) | $\chi_{n=742, df=1}^{2}$ = 0.00, *p* = 0.99 |  |
| CASI (maximum score = 100) | 68.88 (15.99) | 69.57 (16.94) | *t_(df = 749)_* = 0.57, *p* = 0.57 |  |
| HAI-ADL (maximum score = 43) | 8.94 (7.42) | 8.57 (6.71) | *t_(df = 749)_* = 0.71, *p* = 0.48 |  |
| NPI-SB (maximum score = 144) | 7.23 (10.39) | 6.89 (8.68) | *t_(df = 749)_* = 0.49, *p* = 0.63 |  |
| CDR-SB (maximum score = 18) | 5.52 (2.99) | 4.60 (3.16) | *t_(df = 749)_* = 4.07, *p* < 0.001 |  |
| CFS (maximum = 7) | 2.97 (1.76) | 2.95 (1.71) | *t_(df = 749)_* = 0.16, *p* = 0.88 |  |
| TC change | | -30.14 (23.19) | 20.99 (18.92) | *t_(df = 749)_* = 32.99, *p* < 0.001 |
| CASI: Cognitive Assessment Screening Instrument; CDR-SB: Clinical Dementia Rating-Sum of boxes; CFS: Clinical Frailty Scale; Dementia-D: Individuals with progressive dementia; Dementia-S: Individuals with dementia who were not progressive; HAI-ADL: History-Based Artificial Intelligence-Activities of Daily Living; HDL-c: High-density lipoprotein cholesterol; LDL-c: Low-density lipoprotein cholesterol; MCI: Mild cognitive impairment; NPI-SB: Neuropsychiatric Inventory-Sum of boxes; SMCI-D: Subjective or mild cognitive impairment individuals who converted to dementia; SMCI-S: Stable subjective or mild cognitive impairment individuals; TC: Total cholesterol; TG: Triglyceride.  Numbers are denoted as mean (SD) or proportion (number) | | | | |

**Supplementary Table 11.** Demographics and clinical characteristics of the SMCI participants with larger change in serum LDL-c

|  | 1^st^ Quartile | | 4^th^ Quartile | | Statistical comparisons | |  |  |
| --- | --- | --- | --- | --- | --- | --- | --- | --- |
| n | 290 | | 306 | |  | |  |  |
| MCI (%) | 81.03 (235/290) | | 81.04 (248/306) | | $\chi_{n=596, df=1}^{2}$ = 0.00, *p* = 0.99 | |  |  |
| Age (yr) | 74.47 (7.65) | | 74.28 (7.88) | | *t_(df = 594)_* = 0.30, *p* = 0.77 | |  |  |
| Follow-up duration (yr) | 2.76 (1.30) | | 2.86 (1.43) | | *t_(df = 594)_* = 0.89, *p* = 0.37 | |  |  |
| Educational level (yr) | 5.96 (4.37) | | 5.83 (4.54) | | *t_(df = 594)_* = 0.36, *p* = 0.72 | |  |  |
| Sex (% male) | 43.11 (125/290) | | 43.14 (132/290) | | $\chi_{n=596, df=1}^{2}$ = 0.34, *p* = 0.56 | |  |  |
| Hypertension (%) | 72.07 (209/290) | | 77.13 (236/306) | | $\chi_{n=596, df=1}^{2}$ = 2.01, *p* = 0.16 | |  |  |
| Diabetes mellitus (%) | 39.32 (114/290) | | 48.04 (147/306) | | $\chi_{n=596, df=1}^{2}$ = 49.62, *p* < 0.001 | |  |  |
| Coronary artery disease (%) | 12.07 (35/290) | | 15.04 (46/306) | | $\chi_{n=596, df=1}^{2}$ = 2.40, *p* = 0.12 | |  |  |
| Cerebrovascular disease (%) | 43.11 (125/290) | | 42.16 (129/306) | | $\chi_{n=596, df=1}^{2}$ = 0.05, *p* = 0.82 | |  |  |
| Arrhythmia (%) | 12.07 (35/290) | | 17.33 (53/306) | | $\chi_{n=596, df=1}^{2}$ = 3.90, *p* < 0.05 | |  |  |
| Anti-hypertensive (%) | 32.07 (93/290) | | 38.24 (117/306) | | $\chi_{n=596, df=1}^{2}$ = 2.48, *p* = 0.12 | |  |  |
| Anti-diabetic (%) | 56.21 (163/290) | | 56.21 (172/306) | | $\chi_{n=596, df=1}^{2}$ = 0.00, *p* = 0.99 | |  |  |
| Anti-platelets (%) | 56.21 (163/290) | | 56.21 (172/306) | | $\chi_{n=596, df=1}^{2}$ = 0.00, *p* = 0.99 | |  |  |
| Anti-coagulants (%) | 10.00 (29/290) | | 14.06 (43/306) | | $\chi_{n=596, df=1}^{2}$ = 2.30, *p* = 0.13 | |  |  |
| Anti-lipid agents (%) | 45.18 (131/290) | | 38.24 (117/306) | | $\chi_{n=596, df=1}^{2}$ = 2.95, *p* = 0.09 | |  |  |
| CASI (maximum score = 100) | 70.72 (16.76) | | 70.71 (16.16) | | *t_(df = 594)_* = 0.01, *p* = 0.99 | |  |  |
| HAI-ADL (maximum score = 43) | 8.94 (7.42) | | 8.57 (6.71) | | *t_(df = 594)_* = 0.64, *p* = 0.52 | |  |  |
| NPI-SB (maximum score = 144) | 7.23 (10.39) | | 6.89 (8.68) | | *t_(df = 594)_* = 0.43, *p* = 0.66 | |  |  |
| CDR-SB (maximum score = 18) | 2.97 (1.76) | | 2.95 (1.71) | | *t_(df = 594)_* = 0.14, *p* = 0.89 | |  |  |
| CFS (maximum = 7) | 2.98 (1.76) | | 2.95 (1.71) | | *t_(df = 594)_* = 0.21, *p* = 0.83 | |  |  |
| LDL-c change | | | -30.14 (23.19) | | 20.99 (18.92) | | *t_(df = 594)_* = 29.56, *p* < 0.001 | |
| CASI: Cognitive Assessment Screening Instrument; CDR-SB: Clinical Dementia Rating-Sum of boxes; CFS: Clinical Frailty Scale; Dementia-D: Individuals with progressive dementia; Dementia-S: Individuals with dementia who were not progressive; HAI-ADL: History-Based Artificial Intelligence-Activities of Daily Living; HDL-c: High-density lipoprotein cholesterol; LDL-c: Low-density lipoprotein cholesterol; MCI: Mild cognitive impairment; NPI-SB: Neuropsychiatric Inventory-Sum of boxes; SMCI-D: Subjective or mild cognitive impairment individuals who converted to dementia; SMCI-S: Stable subjective or mild cognitive impairment individuals; TC: Total cholesterol; TG: Triglyceride.  Numbers are denoted as mean (SD) or proportion (number) | | | | | | | | |

**Supplementary Table 12.** Demographics and clinical characteristics of the SMCI participants with larger change in serum HDL-c

|  | 1^st^ Quartile | 4^th^ Quartile | Statistical comparisons |  |
| --- | --- | --- | --- | --- |
| n | 337 | 299 |  |  |
| MCI (%) | 85.16 (287/337) | 84.28 (252/299) | $\chi_{n=636, df=1}^{2}$ = 0.29, *p* = 0.59 |  |
| Age (yr) | 75.06 (7.51) | 74.83 (7.81) | *t_(df = 634)_* = 0.38, *p* = 0.71 |  |
| Follow-up duration (yr) | 2.45 (1.36) | 2.29 (1.36) | *t_(df = 634)_* = 1.48, *p* = 0.14 |  |
| Educational level (yr) | 5.64 (4.48) | 5.93 (4.54) | *t_(df = 634)_* = 0.81, *p* = 0.42 |  |
| Sex (% male) | 45.11 (152/337) | 43.15 (129/299) | $\chi_{n=636, df=1}^{2}$ = 0.25, *p* = 0.62 |  |
| Hypertension (%) | 75.08 (253/337) | 73.25 (219/299) | $\chi_{n=636, df=1}^{2}$ = 0.28, *p* = 0.60 |  |
| Diabetes mellitus (%) | 45.11 (152/337) | 40.14 (120/299) | $\chi_{n=636, df=1}^{2}$ = 1.60, *p* = 0.21 |  |
| Coronary artery disease (%) | 11.28 (38/337) | 11.04 (33/299) | $\chi_{n=636, df=1}^{2}$ = 0.01, *p* = 0.92 |  |
| Cerebrovascular disease (%) | 43.03 (145/337) | 42.15 (126/299) | $\chi_{n=636, df=1}^{2}$ = 0.05, *p* = 0.82 |  |
| Arrhythmia (%) | 13.65 (46/337) | 14.05(42/299) | $\chi_{n=636, df=1}^{2}$ = 0.02, *p* = 0.88 |  |
| Anti-hypertensive (%) | 75.08 (253/337) | 73.25 (219/299) | $\chi_{n=636, df=1}^{2}$ = 0.28, *p* = 0.60 |  |
| Anti-diabetic (%) | 34.13 (115/337) | 32.11 (96/299) | $\chi_{n=636, df=1}^{2}$ = 0.29, *p* = 0.59 |  |
| Anti-platelets (%) | 57.28 (193/337) | 56.19 (168/299) | $\chi_{n=636, df=1}^{2}$ = 0.08, *p* = 0.78 |  |
| Anti-coagulants (%) | 11.28 (38/337) | 14.05 (42/299) | $\chi_{n=636, df=1}^{2}$ = 1.11, *p* = 0.29 |  |
| Anti-lipid agents (%) | 37.10 (125/337) | 39.14 (117/299) | $\chi_{n=636, df=1}^{2}$ = 0.28, *p* = 0.60 |  |
| CASI (maximum score = 100) | 70.01 (17.23) | 71.23 (19.19) | *t_(df = 634)_* = 0.85, *p* = 0.40 |  |
| HAI-ADL (maximum score = 43) | 8.94 (7.42) | 8.57 (6.71) | *t_(df = 634)_* = 0.66, *p* = 0.51 |  |
| NPI-SB (maximum score = 144) | 7.23 (10.39) | 6.89 (8.68) | *t_(df = 634)_* = 0.44, *p* = 0.66 |  |
| CDR-SB (maximum score = 18) | 1.78 (1.51) | 1.64 (1.25) | *t_(df = 634)_* = 1.26, *p* = 0.21 |  |
| CFS (maximum = 7) | 2.97 (1.76) | 2.95 (1.71) | *t_(df = 634)_* = 0.15, *p* = 0.89 |  |
| HDL-c change | -30.14 (23.19) | 20.99 (18.92) | *t_(df = 634)_* = 30.24, *p* < 0.001 |  |
| CASI: Cognitive Assessment Screening Instrument; CDR-SB: Clinical Dementia Rating-Sum of boxes; CFS: Clinical Frailty Scale; Dementia-D: Individuals with progressive dementia; Dementia-S: Individuals with dementia who were not progressive; HAI-ADL: History-Based Artificial Intelligence-Activities of Daily Living; HDL-c: High-density lipoprotein cholesterol; LDL-c: Low-density lipoprotein cholesterol; MCI: Mild cognitive impairment; NPI-SB: Neuropsychiatric Inventory-Sum of boxes; SMCI-D: Subjective or mild cognitive impairment individuals who converted to dementia; SMCI-S: Stable subjective or mild cognitive impairment individuals; TC: Total cholesterol; TG: Triglyceride.  Numbers are denoted as mean (SD) or proportion (number) | | | | |

**Supplementary Table 13.** Demographics and clinical characteristics of the SMCI participants with larger change in serum TG

|  | 1^st^ Quartile | 4^th^ Quartile | Statistical comparisons |  |
| --- | --- | --- | --- | --- |
| n | 328 | 341 |  |  |
| MCI (%) | 84.25 (246/328) | 88.00 (286/341) | $\chi_{n=669, df=1}^{2}$ = 8.08, *p* < 0.01 |  |
| Age (yr) | 74.70 (7.40) | 74.52 (7.57) | *t_(df = 667)_* = 0.31, *p* = 0.76 |  |
| Follow-up duration (yr) | 2.88 (1.45) | 2.67 (1.37) | *t_(df = 667)_* = 1.93, *p* = 0.05 |  |
| Educational level (yr) | 5.67 (4.37) | 5.86 (4.52) | *t_(df = 667)_* = 0.55, *p* = 0.58 |  |
| Sex (% male) | 43.30 (142/328) | 47.22 (161/341) | $\chi_{n=669, df=1}^{2}$ = 1.04, *p* = 0.31 |  |
| Hypertension (%) | 78.05 (256/328) | 74.20 (253/341) | $\chi_{n=669, df=1}^{2}$ = 1.37, *p* = 0.24 |  |
| Diabetes mellitus (%) | 50.00 (164/328) | 50.15 (164/341) | $\chi_{n=669, df=1}^{2}$ = 0.001, *p* = 0.97 |  |
| Coronary artery disease (%) | 11.29 (37/328) | 12.03 (37/341) | $\chi_{n=669, df=1}^{2}$ = 0.09, *p* = 0.76 |  |
| Cerebrovascular disease (%) | 31.10 (102/328) | 30.21 (102/341) | $\chi_{n=669, df=1}^{2}$ = 0.06, *p* = 0.80 |  |
| Arrhythmia (%) | 14.03 (46/328) | 16.13 (46/341) | $\chi_{n=669, df=1}^{2}$ = 0.58, *p* = 0.45 |  |
| Anti-hypertensive (%) | 78.05 (256/328) | 75.08 (256/341) | $\chi_{n=669, df=1}^{2}$ = 0.82, *p* = 0.36 |  |
| Anti-diabetic (%) | 35.07 (115/328) | 38.13 (115/341) | $\chi_{n=669, df=1}^{2}$ = 0.67, *p* = 0.41 |  |
| Anti-platelets (%) | 56.10 (184/328) | 60.12 (184/341) | $\chi_{n=669, df=1}^{2}$ = 1.11, *p* = 0.29 |  |
| Anti-coagulants (%) | 12.20 (40/328) | 15.25 (40/341) | $\chi_{n=669, df=1}^{2}$ = 1.31, *p* = 0.25 |  |
| Anti-lipid agents (%) | 38.11 (125/328) | 37.25 (127/341) | $\chi_{n=669, df=1}^{2}$ = 0.05, *p* = 0.82 |  |
| CASI (maximum score = 100) | 70.23 (17.42) | 68.92 (19.40) | *t_(df = 667)_* = 0.92, *p* = 0.36 |  |
| HAI-ADL (maximum score = 43) | 3.92 (3.10) | 3.55 (2.85) | *t_(df = 667)_* = 1.61, *p* = 0.11 |  |
| NPI-SB (maximum score = 144) | 4.39 (5.79) | 4.73 (6.15) | *t_(df = 667)_* = 0.74, *p* = 0.46 |  |
| CDR-SB (maximum score = 18) | 1.65 (1.25) | 1.58 (1.41) | *t_(df = 667)_* = 0.68, *p* = 0.50 |  |
| CFS (maximum = 7) | 2.80 (1.60) | 2.92 (1.44) | *t_(df = 667)_* = 1.02, *p* = 0.31 |  |
| TG change | -69.24 (88.44) | 39.54 (46.85) | *t_(df = 667)_* = 19.99, *p* < 0.001 |  |
| CASI: Cognitive Assessment Screening Instrument; CDR-SB: Clinical Dementia Rating-Sum of boxes; CFS: Clinical Frailty Scale; Dementia-D: Individuals with progressive dementia; Dementia-S: Individuals with dementia who were not progressive; HAI-ADL: History-Based Artificial Intelligence-Activities of Daily Living; HDL-c: High-density lipoprotein cholesterol; LDL-c: Low-density lipoprotein cholesterol; MCI: Mild cognitive impairment; NPI-SB: Neuropsychiatric Inventory-Sum of boxes; SMCI-D: Subjective or mild cognitive impairment individuals who converted to dementia; SMCI-S: Stable subjective or mild cognitive impairment individuals; TC: Total cholesterol; TG: Triglyceride.  Numbers are denoted as mean (SD) or proportion (number) | | | | |

**Supplementary Table 14.** Demographics and clinical characteristics of the participants with dementia and larger change in serum TC

|  | 1^st^ Quartile | 4^th^ Quartile | Statistical comparisons |  |
| --- | --- | --- | --- | --- |
| n | 209 | 169 |  |  |
| Age (yr) | 80.13 (7.63) | 78.97 (7.44) | *t_(df = 276)_* = 1.49, *p* = 0.14 |  |
| Follow-up duration (yr) | 2.55 (1.72) | 2.68 (1.75) | *t_(df = 276)_* = 0.73, *p* = 0.47 |  |
| Educational level (yr) | 4.45 (4.33) | 4.12 (4.25) | *t_(df = 276)_* = 0.74, *p* = 0.46 |  |
| Sex (% male) | 48.24 (123/209) | 28.09 (66/169) | $\chi_{n=278, df=1}^{2}$ = 20.96, *p* < 0.001 |  |
| Hypertension (%) | 73.34 (187/209) | 74.05 (174/169) | $\chi_{n=278, df=1}^{2}$ = 0.03, *p* = 0.86 |  |
| Diabetes mellitus (%) | 57.26 (146/209) | 38.30 (90/169) | $\chi_{n=278, df=1}^{2}$ = 17.60, *p* < 0.001 |  |
| Coronary artery disease (%) | 12.16 (31/209) | 8.09 (19/169) | $\chi_{n=278, df=1}^{2}$ = 2.21, *p* = 0.14 |  |
| Cerebrovascular disease (%) | 49.02 (125/209) | 47.24 (111/169) | $\chi_{n=278, df=1}^{2}$ = 0.16, *p* = 0.69 |  |
| Arrhythmia (%) | 17.26 (44/209) | 11.07 (26/169) | $\chi_{n=278, df=1}^{2}$ = 3.83, *p* = 0.05 |  |
| Anti-hypertensive (%) | 70.20 (179/209) | 72.35 (170/169) | $\chi_{n=278, df=1}^{2}$ = 0.27, *p* = 0.60 |  |
| Anti-diabetic (%) | 44.32 (113/209) | 30.22 (71/169) | $\chi_{n=278, df=1}^{2}$ = 10.37, *p* < 0.01 |  |
| Anti-platelets (%) | 57.26 (146/209) | 51.07 (120/169) | $\chi_{n=278, df=1}^{2}$ = 1.89, *p* = 0.17 |  |
| Anti-coagulants (%) | 16.08 (41/209) | 9.37 (22/169) | $\chi_{n=278, df=1}^{2}$ = 4.92, *p* < 0.05 |  |
| Anti-lipid agents (%) | 47.06 (120/209) | 49.37 (116/169) | $\chi_{n=278, df=1}^{2}$ = 0.26, *p* = 0.61 |  |
| CASI (maximum score = 100) | 42.16 (19.41) | 42.79 (19.31) | *t_(df = 276)_* = 0.32, *p* = 0.75 |  |
| HAI-ADL (maximum score = 43) | 16.17 (5.65) | 15.69 (5.76) | *t_(df = 276)_* = 0.81, *p* = 0.42 |  |
| NPI-SB (maximum score = 144) | 10.20 (11.64) | 10.88 (11.26) | *t_(df = 276)_* = 0.57, *p* = 0.57 |  |
| CDR-SB (maximum score = 18) | 8.28 (2.95) | 8.29 (3.15) | *t_(df = 276)_* = 0.03, *p* = 0.98 |  |
| CFS (maximum = 7) | 4.35 (1.40) | 4.38 (1.45) | *t_(df = 276)_* = 0.23, *p* = 0.82 |  |
| TC change | -31.36 (22.88) | 21.81 (19.54) | *t_(df = 276)_* = 27.66, *p* < 0.001 |  |
| CASI: Cognitive Assessment Screening Instrument; CDR-SB: Clinical Dementia Rating-Sum of boxes; CFS: Clinical Frailty Scale; Dementia-D: Individuals with progressive dementia; Dementia-S: Individuals with dementia who were not progressive; HAI-ADL: History-Based Artificial Intelligence-Activities of Daily Living; HDL-c: High-density lipoprotein cholesterol; LDL-c: Low-density lipoprotein cholesterol; MCI: Mild cognitive impairment; NPI-SB: Neuropsychiatric Inventory-Sum of boxes; SMCI-D: Subjective or mild cognitive impairment individuals who converted to dementia; SMCI-S: Stable subjective or mild cognitive impairment individuals; TC: Total cholesterol; TG: Triglyceride.  Numbers are denoted as mean (SD) or proportion (number) | | | | |

**Supplementary Table 15.** Demographics and clinical characteristics of the participants with dementia and larger change in serum LDL-c

|  | 1^st^ Quartile | 4^th^ Quartile | Statistical comparisons |  |
| --- | --- | --- | --- | --- |
| n | 194 | 190 |  |  |
| Age (yr) | 78.40 (7.47) | 78.56 (7.21) | *t_(df = 382)_* = 0.21, *p* = 0.83 |  |
| Follow-up duration (yr) | 2.86 (1.38) | 2.72 (1.34) | *t_(df = 382)_* = 1.01, *p* = 0.31 |  |
| Educational level (yr) | 4.19 (4.17) | 3.91 (4.07) | *t_(df = 382)_* = 0.67, *p* = 0.51 |  |
| Sex (% male) | 38 (74/194) | 42 (80/190) | $\chi_{n=384, df=1}^{2}$ = 0.63, *p* = 0.43 |  |
| Hypertension (%) | 77 (150/194) | 74 (141/190) | $\chi_{n=384, df=1}^{2}$ = 0.51, *p* = 0.48 |  |
| Diabetes mellitus (%) | 55 (107/194) | 56 (107/190) | $\chi_{n=384, df=1}^{2}$ = 0.05, *p* = 0.82 |  |
| Coronary artery disease (%) | 9 (18/194) | 11 (18/190) | $\chi_{n=384, df=1}^{2}$ = 0.33, *p* = 0.56 |  |
| Cerebrovascular disease (%) | 50 (97/194) | 53 (97/190) | $\chi_{n=384, df=1}^{2}$ = 0.38, *p* = 0.54 |  |
| Arrhythmia (%) | 15 (30/194) | 11 (30/190) | $\chi_{n=384, df=1}^{2}$ = 1.62, *p* = 0.20 |  |
| Anti-hypertensive (%) | 47 (92/194) | 44 (92/190) | $\chi_{n=384, df=1}^{2}$ = 0.40, *p* = 0.53 |  |
| Anti-diabetic (%) | 64 (125/194) | 55 (125/190) | $\chi_{n=384, df=1}^{2}$ = 3.36, *p* = 0.07 |  |
| Anti-platelets (%) | 64 (125/194) | 55 (125/190) | $\chi_{n=384, df=1}^{2}$ = 3.36, *p* = 0.07 |  |
| Anti-coagulants (%) | 15 (30/194) | 11 (30/190) | $\chi_{n=384, df=1}^{2}$ = 1.62, *p* = 0.20 |  |
| Anti-lipid agents (%) | 36 (70/194) | 30 (70/190) | $\chi_{n=384, df=1}^{2}$ = 1.60, *p* = 0.21 |  |
| CASI (maximum score = 100) | 43.38 (19.60) | 43.08 (19.62) | *t_(df = 382)_* = 0.15, *p* = 0.88 |  |
| HAI-ADL (maximum score = 43) | 16.26 (5.84) | 16.15 (5.79) | *t_(df = 382)_* = 0.19, *p* = 0.85 |  |
| NPI-SB (maximum score = 144) | 10.27 (11.35) | 10.80 (12.01) | *t_(df = 382)_* = 0.45, *p* = 0.66 |  |
| CDR-SB (maximum score = 18) | 9.21 (3.82) | 9.00 (3.72) | *t_(df = 382)_* = 0.55, *p* = 0.59 |  |
| CFS (maximum = 7) | 4.36 (1.44) | 4.45 (1.47) | *t_(df = 382)_* = 0.60, *p* = 0.55 |  |
| LDL-c change | -30.07 (19.86) | 23.26 (18.29) | *t_(df = 382)_* = 27.36, *p* < 0.001 |  |
| CASI: Cognitive Assessment Screening Instrument; CDR-SB: Clinical Dementia Rating-Sum of boxes; CFS: Clinical Frailty Scale; Dementia-D: Individuals with progressive dementia; Dementia-S: Individuals with dementia who were not progressive; HAI-ADL: History-Based Artificial Intelligence-Activities of Daily Living; HDL-c: High-density lipoprotein cholesterol; LDL-c: Low-density lipoprotein cholesterol; MCI: Mild cognitive impairment; NPI-SB: Neuropsychiatric Inventory-Sum of boxes; SMCI-D: Subjective or mild cognitive impairment individuals who converted to dementia; SMCI-S: Stable subjective or mild cognitive impairment individuals; TC: Total cholesterol; TG: Triglyceride.  Numbers are denoted as mean (SD) or proportion (number) | | | | |

**Supplementary Table 16.** Demographics and clinical characteristics of the participants with dementia and larger change in serum HDL-c

|  | 1^st^ Quartile | 4^th^ Quartile | Statistical comparisons |  |
| --- | --- | --- | --- | --- |
| n | 228 | 201 |  |  |
| Age (yr) | 78.96 (7.17) | 79.01 (7.41) | *t_(df = 427)_* = 0.07, *p* = 0.94 |  |
| Follow-up duration (yr) | 2.94 (1.44) | 2.67 (1.40) | *t_(df = 427)_* = 1.96, *p* = 0.05 |  |
| Educational level (yr) | 4.32 (4.30) | 4.84 (4.30) | *t_(df = 427)_* = 1.25, *p* = 0.21 |  |
| Sex (% male) | 36.41 (83/228) | 42.29 (85/201) | $\chi_{n=429,df=1}^{2}$ = 1.55, *p* = 0.21 |  |
| Hypertension (%) | 76.32 (174/228) | 73.14 (147/201) | $\chi_{n=429,df=1}^{2}$ = 0.57, *p* = 0.45 |  |
| Diabetes mellitus (%) | 49.13 (112/228) | 50.25 (101/201) | $\chi_{n=429,df=1}^{2}$ = 0.05, *p* = 0.82 |  |
| Coronary artery disease (%) | 6.15 (14/228) | 11.45 (23/201) | $\chi_{n=429,df=1}^{2}$ = 3.81, *p* = 0.05 |  |
| Cerebrovascular disease (%) | 30.27 (69/228) | 31.35 (63/201) | $\chi_{n=429,df=1}^{2}$ = 0.06, *p* = 0.81 |  |
| Arrhythmia (%) | 12.29 (28/228) | 12.44 (25/201) | $\chi_{n=429,df=1}^{2}$ = 0.00, *p* = 0.96 |  |
| Anti-hypertensive (%) | 75.00 (171/228) | 69.16 (139/201) | $\chi_{n=429,df=1}^{2}$ = 1.82, *p* = 0.18 |  |
| Anti-diabetic (%) | 38.16 (87/228) | 39.31 (79/201) | $\chi_{n=429,df=1}^{2}$ = 0.06, *p* = 0.81 |  |
| Anti-platelets (%) | 62.29 (142/228) | 54.23 (109/201) | $\chi_{n=429,df=1}^{2}$ = 2.85, *p* = 0.09 |  |
| Anti-coagulants (%) | 10.09 (23/228) | 13.44 (27/201) | $\chi_{n=429,df=1}^{2}$ = 1.16, *p* = 0.28 |  |
| Anti-lipid agents (%) | 30.27 (69/228) | 27.37 (55/201) | $\chi_{n=429,df=1}^{2}$ = 0.43, *p* = 0.51 |  |
| CASI (maximum score = 100) | 42.01 (18.20) | 44.55 (19.40) | *t_(df = 427)_* = 1.40, *p* = 0.16 |  |
| HAI-ADL (maximum score = 43) | 4.35 (3.46) | 4.00 (3.03) | *t_(df = 427)_* = 1.11, *p* = 0.27 |  |
| NPI-SB (maximum score = 144) | 4.56 (6.64) | 4.48 (5.76) | *t_(df = 427)_* = 0.13, *p* = 0.90 |  |
| CDR-SB (maximum score = 18) | 1.78 (1.51) | 1.64 (1.25) | *t_(df = 427)_* = 1.04, *p* = 0.30 |  |
| CFS (maximum = 7) | 2.00 (1.07) | 1.86 (1.01) | *t_(df = 427)_* = 1.39, *p* = 0.17 |  |
| HDL-c change | -8.60 (8.46) | 9.38 (8.74) | *t_(df = 427)_* = 21.64, *p* < 0.001 |  |
| CASI: Cognitive Assessment Screening Instrument; CDR-SB: Clinical Dementia Rating-Sum of boxes; CFS: Clinical Frailty Scale; Dementia-D: Individuals with progressive dementia; Dementia-S: Individuals with dementia who were not progressive; HAI-ADL: History-Based Artificial Intelligence-Activities of Daily Living; HDL-c: High-density lipoprotein cholesterol; LDL-c: Low-density lipoprotein cholesterol; MCI: Mild cognitive impairment; NPI-SB: Neuropsychiatric Inventory-Sum of boxes; SMCI-D: Subjective or mild cognitive impairment individuals who converted to dementia; SMCI-S: Stable subjective or mild cognitive impairment individuals; TC: Total cholesterol; TG: Triglyceride.  Numbers are denoted as mean (SD) or proportion (number) | | | | |

**Supplementary Table 17.** Demographics and clinical characteristics of the participants with dementia and larger change in serum TG

|  | 1^st^ Quartile | 4^th^ Quartile | Statistical comparisons |  |
| --- | --- | --- | --- | --- |
| n | 236 | 217 |  |  |
| Age (yr) | 79.82 (7.36) | 78.69 (7.34) | *t_(df = 451)_* = 1.64, *p* = 0.10 |  |
| Follow-up duration (yr) | 2.89 (1.46) | 2.76 (1.46) | *t_(df = 451)_* = 0.95, *p* = 0.34 |  |
| Educational level (yr) | 4.58 (4.59) | 4.36 (4.50) | *t_(df = 451)_* = 0.51, *p* = 0.61 |  |
| Sex (% male) | 40.26 (95/236) | 42.86 (93/217) | $\chi_{n=453,df=1}^{2}$ = 0.32, *p* = 0.57 |  |
| Hypertension (%) | 76.28 (180/236) | 81.57 (177/217) | $\chi_{n=453,df=1}^{2}$ = 1.90, *p* = 0.17 |  |
| Diabetes mellitus (%) | 59.75 (141/236) | 59.91 (130/217) | $\chi_{n=453,df=1}^{2}$ = 0.00, *p* = 0.97 |  |
| Coronary artery disease (%) | 9.33 (22/236) | 10.14 (22/217) | $\chi_{n=453,df=1}^{2}$ = 0.09, *p* = 0.77 |  |
| Cerebrovascular disease (%) | 31.36 (74/236) | 32.72 (71/217) | $\chi_{n=453,df=1}^{2}$ = 0.10, *p* = 0.76 |  |
| Arrhythmia (%) | 14.41 (34/236) | 14.29 (31/217) | $\chi_{n=453,df=1}^{2}$ = 0.00, *p* = 0.97 |  |
| Anti-hypertensive (%) | 72.04 (170/236) | 80.65 (175/217) | $\chi_{n=453,df=1}^{2}$ = 4.62, *p* < 0.05 |  |
| Anti-diabetic (%) | 49.16 (116/236) | 47.93 (104/217) | $\chi_{n=453,df=1}^{2}$ = 0.07, *p* = 0.79 |  |
| Anti-platelets (%) | 56.36 (133/236) | 65.44 (142/217) | $\chi_{n=453,df=1}^{2}$ = 3.91, *p* < 0.05 |  |
| Anti-coagulants (%) | 13.14 (31/236) | 13.37 (29/217) | $\chi_{n=453,df=1}^{2}$ = 0.01, *p* = 0.94 |  |
| Anti-lipid agents (%) | 29.24 (69/236) | 30.88 (67/217) | $\chi_{n=453,df=1}^{2}$ = 0.14, *p* = 0.70 |  |
| CASI (maximum score = 100) | 43.67 (17.62) | 44.98 (19.23) | *t_(df = 451)_* = 0.76, *p* = 0.45 |  |
| HAI-ADL (maximum score = 43) | 17.06 (7.05) | 16.96 (6.76) | *t_(df = 451)_* = 0.15, *p* = 0.88 |  |
| NPI-SB (maximum score = 144) | 10.72 (11.99) | 12.61 (12.38) | *t_(df = 451)_* = 1.65, *p* = 0.10 |  |
| CDR-SB (maximum score = 18) | 9.21 (3.82) | 9.00 (3.72) | *t_(df = 451)_* = 0.59, *p* = 0.55 |  |
| CFS (maximum = 7) | 2.02 (1.00) | 1.90 (0.99) | *t_(df = 451)_* = 1.28, *p* = 0.20 |  |
| TG change | -58.42 (62.55) | 55.37 (55.44) | *t_(df = 451)_* = 20.42, *p* < 0.001 |  |
| CASI: Cognitive Assessment Screening Instrument; CDR-SB: Clinical Dementia Rating-Sum of boxes; CFS: Clinical Frailty Scale; Dementia-D: Individuals with progressive dementia; Dementia-S: Individuals with dementia who were not progressive; HAI-ADL: History-Based Artificial Intelligence-Activities of Daily Living; HDL-c: High-density lipoprotein cholesterol; LDL-c: Low-density lipoprotein cholesterol; MCI: Mild cognitive impairment; NPI-SB: Neuropsychiatric Inventory-Sum of boxes; SMCI-D: Subjective or mild cognitive impairment individuals who converted to dementia; SMCI-S: Stable subjective or mild cognitive impairment individuals; TC: Total cholesterol; TG: Triglyceride.  Numbers are denoted as mean (SD) or proportion (number) | | | | |

**Supplementary Table 18.** Hazards of incident dementia or cognitive progression among the participants with larger change in cholesterol

|  | SMCI | | | Dementia | | |
| --- | --- | --- | --- | --- | --- | --- |
|  | 1st Quartile | 4th Quartile | Statistical comparison | 1st Quartile | 4th Quartile | Statistical comparison |
| TC | **1.69 [1.40 – 2.51]** | **1.34 [1.04 – 3.59]** | **Z = 4.16, *p* < 0.001** | **1.42 [1.26 – 1.78]** | **1.27 [0.81 – 1.34]** | **Z = 6.87, *p* < 0.001** |
| TC change | 1.82 [1.16 – 5.55] | 2.16 [1.18 – 4,86] | Z = 1.81, *p* = 0.07 | 1.56 [1.18 – 1.60] | 1.60 [1.10 – 3.89] | Z = 0.69, *p* = 0.49 |
| LDL-c | 1.51 [1.25 – 2.17] | 1.36 [1.10 – 3.43] | Z = 1.46, *p* = 0.14 | **1.47 [1.20 – 2.56]** | **0.91 [0.90 – 0.94]** | **Z = 7.98, *p* < 0.001** |
| LDL-c change | 1.80 [0.21 – 2.37] | 1.88 [1.14 – 5.02] | Z = 1.43, *p* = 0.15 | 1.72 [1.15 – 2.62] | 1.68 [1.15 – 5.31] | Z = 1.16, *p* = 0.25 |
| HDL | **1.25 [0.99 – 5.64]** | **1.35 [1.24 – 1.58]** | **Z = 3.50, *p* < 0.001** | 0.88 [0.87 – 0.92] | 0.89 [0.87 – 1.26] | Z = 1.82, *p* = 0.07 |
| HDL-c change | 1.15 [1.15 – 5.14] | 1.03 [0.85 –5.89] | Z = 1.45, *p* = 0.14 | **1.64 [1.10 – 4.81]** | **1.28 [0.01 – 1.98]** | **Z = 5.40, *p* < 0.001** |
| TG | **1.16 [1.00 – 1.71]** | **1.03 [0.99 – 2.12]** | **Z = 5.39, *p* < 0.001** | **1.28 [1.10 – 1.98]** | **0.96 [0.95 – 1.79]** | **Z = 7.21, *p* < 0.001** |
| TG change | **1.53 [0.10 – 2.50]** | **1.43 [0.74 – 1.68]** | **Z = 3.72, *p* < 0.001** | **1.68 [0.59 – 2.50]** | **0.93 [0.92 – 10.00]** | **Z = 10.32, *p* < 0.001** |

*Note.* Bold texts denote for statistically significant difference. *Abbreviations:* HDL-c: High-density lipoprotein cholesterol; LDL-c: Low-density lipoprotein cholesterol; SMCI: Subjective or mild cognitive impairment; TC: Total cholesterol; TG: Triglyceride


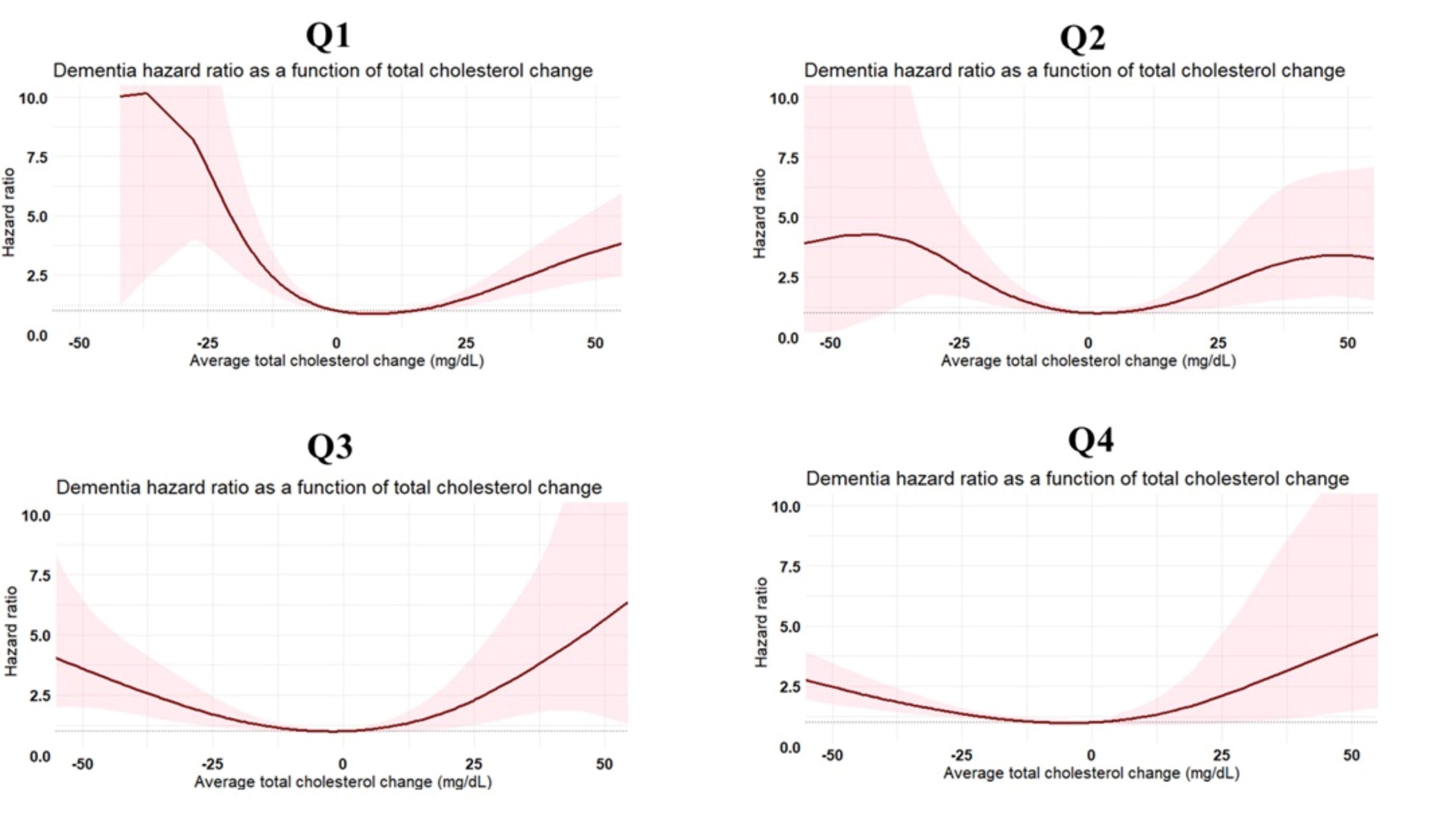


**Supplementary Figure 1.** Association between longitudinal total cholesterol change and dementia risk across baseline total cholesterol quartiles in the SMCI cohort


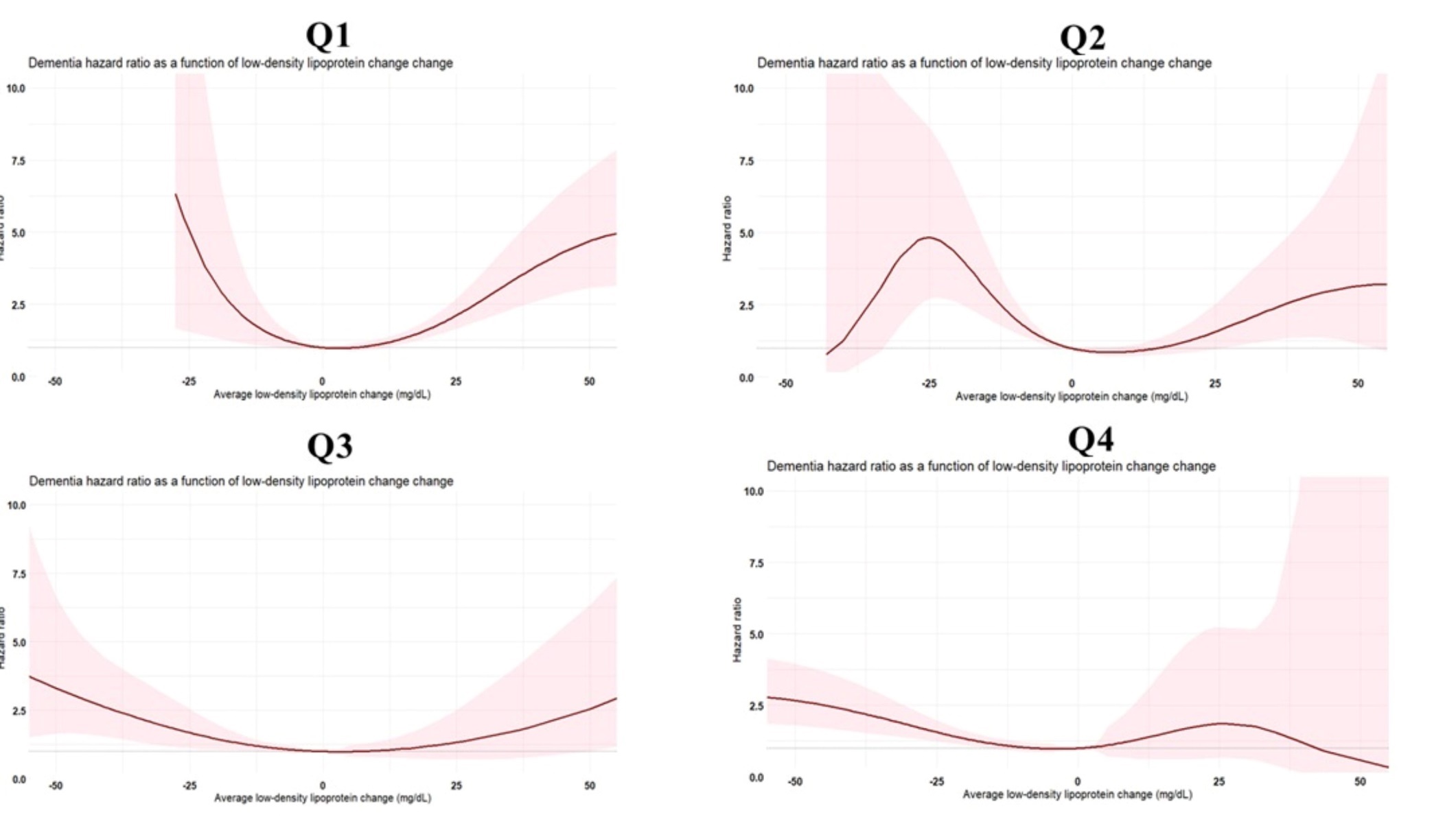


**Supplementary Figure 2.** Association between longitudinal total low-density lipoprotein cholesterol change and dementia risk across baseline total cholesterol quartiles in the SMCI cohort


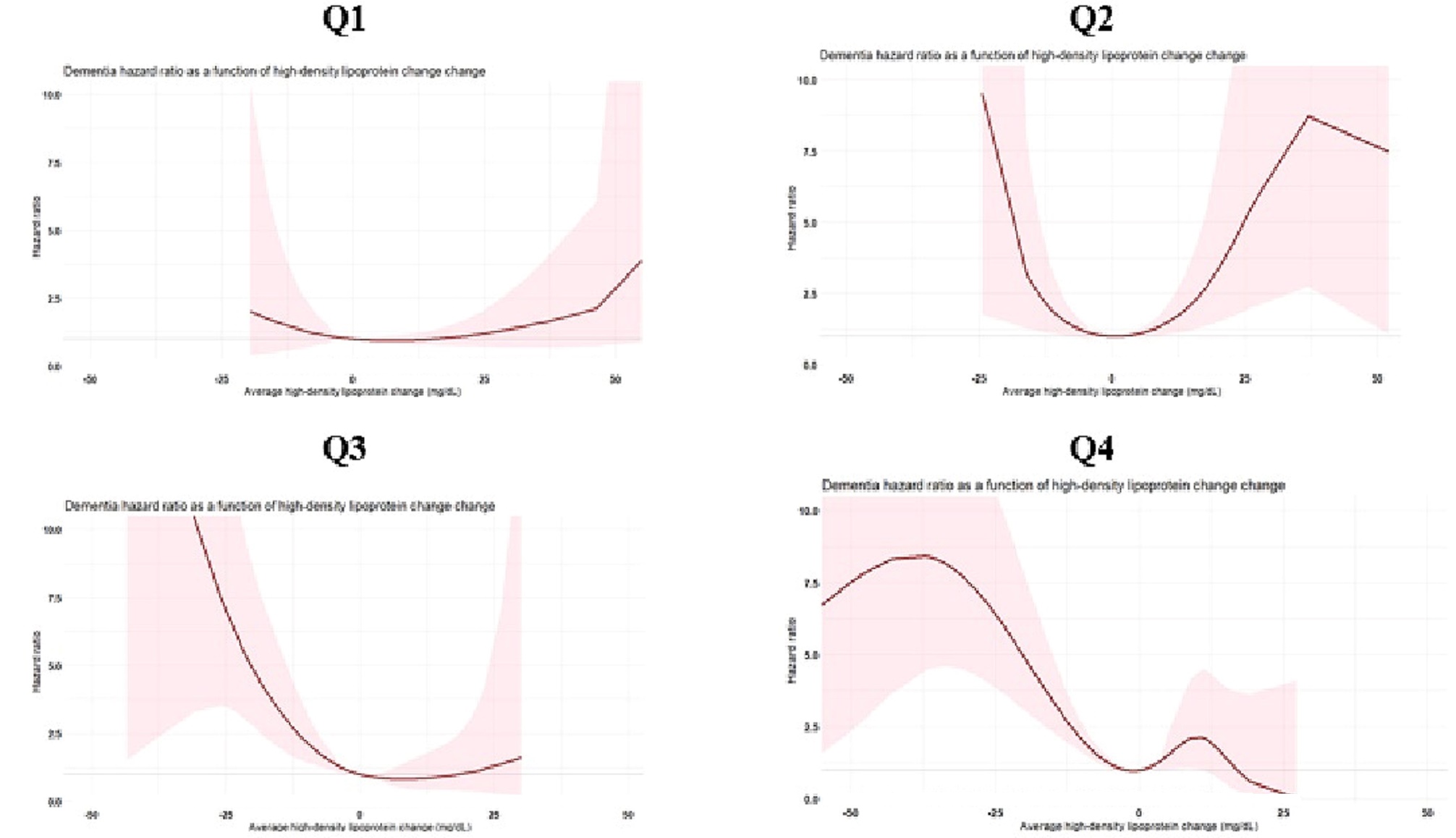


**Supplementary Figure 3.** Association between longitudinal total high-density lipoprotein cholesterol change and dementia risk across baseline total cholesterol quartiles in the SMCI cohort


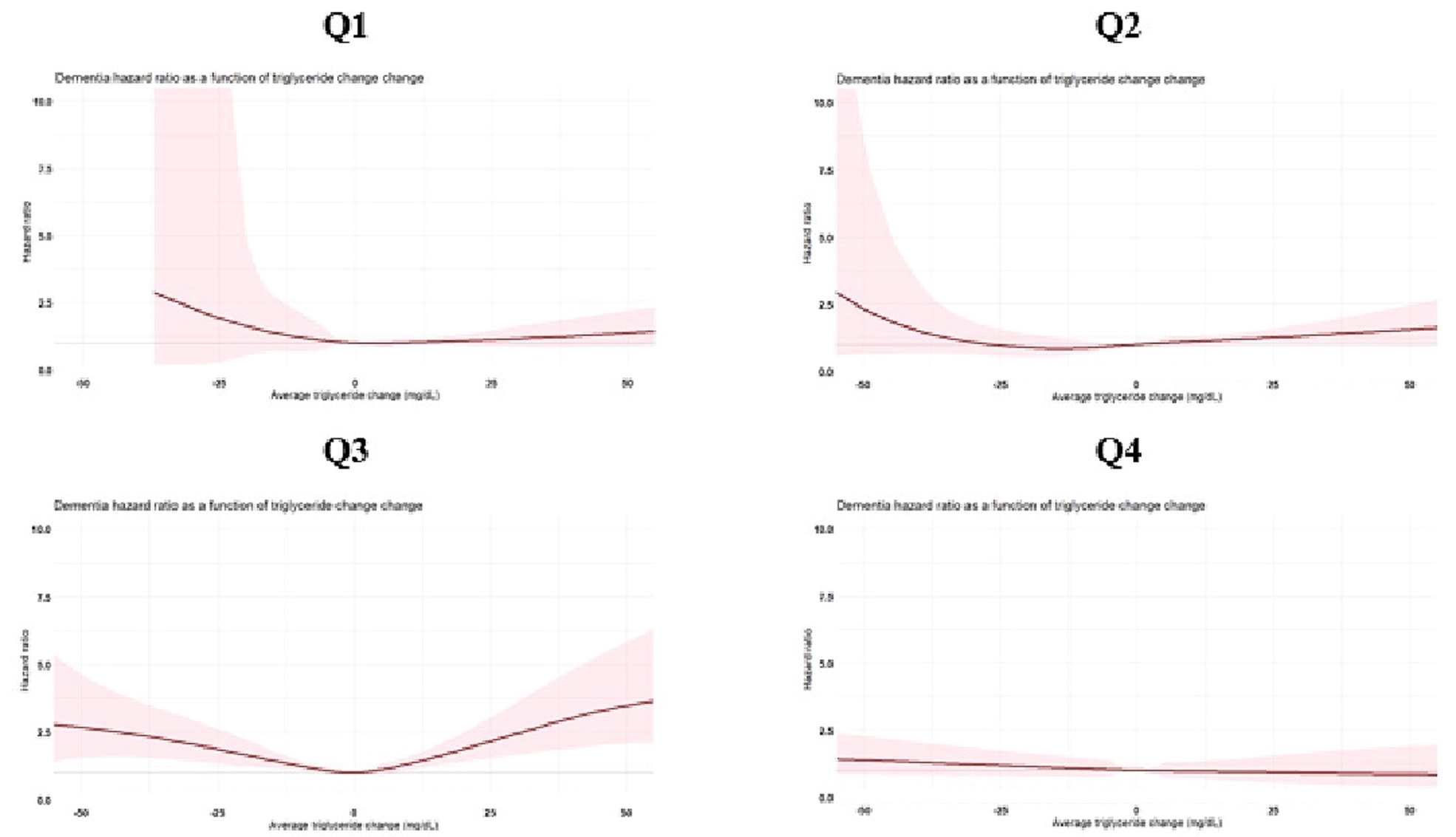


**Supplementary Figure 4.** Association between longitudinal triglyceride change and dementia risk across baseline total cholesterol quartiles in the SMCI cohort
